# Evanescent waves modulate energy efficiency of photocatalysis within TiO_2_ coated optical fibers illuminated using LEDs

**DOI:** 10.1038/s41467-021-24370-8

**Published:** 2021-07-02

**Authors:** Yinghao Song, Li Ling, Paul Westerhoff, Chii Shang

**Affiliations:** 1 Department of Civil and Environmental Engineering, The Hong Kong University of Science and Technology, Hong Kong, China; 2 School of Sustainable Engineering and the Built Environment and Nanosystems Engineering Research Center for Nanotechnology-Enabled Water Treatment (NEWT), Arizona State University, Tempe, AZ USA; 3 Hong Kong Branch of Chinese National Engineering Research Center for Control & Treatment of Heavy Metal Pollution, The Hong Kong University of Science and Technology, Hong Kong, China

**Keywords:** Pollution remediation, Chemical engineering, Photocatalysis, Nanoparticles

## Abstract

Coupling photocatalyst-coated optical fibers (P-OFs) with LEDs shows potential in environmental applications. Here we report a strategy to maximize P-OF light usage and quantify interactions between two forms of light energy (refracted light and evanescent waves) and surface-coated photocatalysts. Different TiO_2_-coated quartz optical fibers (TiO_2_-QOFs) are synthesized and characterized. An energy balance model is then developed by correlating different nano-size TiO_2_ coating structures with light propagation modes in TiO_2_-QOFs. By reducing TiO_2_ patchiness on optical fibers to 0.034 cm^2^/cm^2^ and increasing the average interspace distance between fiber surfaces and TiO_2_ coating layers to 114.3 nm, refraction is largely reduced when light is launched into TiO_2_-QOFs, and 91% of light propagated on the fiber surface is evanescent waves. 24% of the generated evanescent waves are not absorbed by nano-TiO_2_ and returned to optical fibers, thus increasing the quantum yield during degradation of a refractory pollutant (carbamazepine) in water by 32%. Our model also predicts that extending the TiO_2_-QOF length could fully use the returned light to double the carbamazepine degradation and quantum yield. Therefore, maximizing evanescent waves to activate photocatalysts by controlling photocatalyst coating structures emerges as an effective strategy to improve light usage in photocatalysis.

## Abstract (web summary)

Coupling photocatalyst-coated optical fibers (P-OFs) with LEDs shows potential in environmental applications. Here the authors report a strategy to maximize P-OF light usage and simultaneously establish a new platform to quantify photocatalytic performance.

## Introduction

Heterogeneous photocatalysis has shown tremendous potential for solving environmental problems and reducing the energy crisis in the past few decades. Its applications include pollutant degradation, bacteria, and virus inactivation, water splitting for energy production, organic transformations, and more^1–6^. Upon absorption of light, photocatalysts generate hole-electron (h^+^-e^−^) pairs that subsequently undergo redox reactions with molecules adsorbed on photocatalyst surfaces^7–9^. However, current reactor configurations compromise the viability of photocatalytic processes because both slurry and fixed-bed reactors scatter and occlude light, consequently reducing the overall system energy efficiency^10–13^.

Launching light from energy-efficient and low-cost LEDs into optical fibers that are coated with photocatalysts is a new photocatalytic reactor design^14–18^. Such design shows dramatic improvements in quantum yields during pollutant degradation (i.e., moles of pollutants degraded per mole of photons absorbed by photocatalysts). As light propagates along photocatalyst-coated optical fibers (P-OFs), a fraction of the light is refracted from the lower refractive index optical fibers into the higher refractive index surface-coated photocatalyst layers (Supplementary Note 1). This produces reactive oxygen species (ROS), which degrade pollutants at the interface between photocatalysts and water containing the pollutants^17,19,20^. This configuration minimizes light loss due to scattering and occlusion and nearly doubles quantum yields of degradation of three pollutants when compared against slurry reactor systems at equivalent photocatalyst masses^17,20,21^. However, due to thick and dense photocatalyst layers dip-coated on optical fibers using highly concentrated suspension of photocatalysts in previous reports, most light delivered to the P-OFs refracts out of the fiber near the beginning sections of the coating^19,20,22,23^. As such, the majority of the refracted light escaped from the P-OFs without activating photocatalysts and lost its energy in water. Maximizing refracted light absorption by coating more photocatalysts improves light utilization, but thicker and more dense photocatalyst coating layers limited mass transfer of pollutants from water to ROS-producing sites^24^. The resulting quantum yields were thus not enhanced^20^. To realize the potential of reactor designs that can efficiently leverage LED technological advances, P-OFs achieving higher light utilization without compromising photocatalytic performance need to be developed.

To date, P-OF research has focused on managing refracted light. However, light has two energy forms when propagating along P-OFs: refracted light and evanescent waves. We hypothesized that maximizing evanescent wave energy, which is generated during total internal reflection (TIR) (details in Supplementary Note 2) is a better strategy to activate surface-coated photocatalysts than managing refracted light energy. Unlike refracted light, which propagates away from optical fibers and loses its energy in water, evanescent waves propagate on optical fiber surfaces. Evanescent wave energy that does not react with the photocatalyst returns and continues to propagate along the optical fiber where it can subsequently react with photocatalysts located further along the axial path of the optical fiber^25,26^. Thus, light reacts more efficiently with surface-coated photocatalysts through evanescent waves than refracted light.

**Fig. 1:**
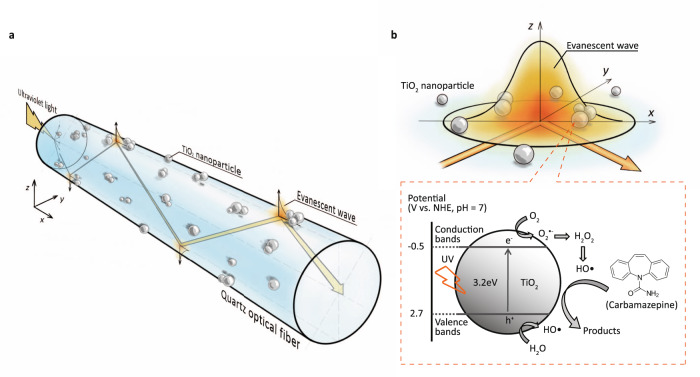
Schematics of the TiO_2_-QOFs with tunable surface “patches” of TiO_2_ layers. **a** Light propagates along the TiO_2_-QOFs and generates evanescent waves when light strikes the optical fiber surfaces without TiO_2_ nanoparticles; and **b** TiO_2_ nanoparticle activation by the generated evanescent waves, and the subsequent production of hydroxyl radicals (HO•) to degrade carbamazepine.

In this work, to test our hypothesis, we evaluate the ability of TiO_2_-coated quartz optical fibers (TiO_2_-QOFs), which have novel tunable surface “patches” of TiO_2_ layers, to promote evanescent wave generation to activate TiO_2_ (Fig. 1a) and degrade a refractory pollutant (carbamazepine) by photocatalytic generated hydroxyl radicals (HO•) in bulk solution and pores in TiO_2_ coating layers (Fig. 1b). Compared with 6.5-cm TiO_2_-QOFs coated with thick and dense TiO_2_ layers, the 6.5-cm TiO_2_-QOFs coated with lower TiO_2_ patchiness increase quantum yield by 32% without compromising carbamazepine degradation efficiency. The TiO_2_-QOFs coated with low TiO_2_ patchiness are fabricated by dip-coating 4.8 μg/cm^2^ nano-TiO_2_ (P25, anatase/rutile 85/15, particle size 21 nm), which is selected for its wide use^7^, on the surfaces of quartz optical fibers, which are selected for their high UV transmission^27^. This coating strategy leaves only 3.4% of the optical fiber surface in direct contact with TiO_2_ and creates 114.3 nm, on-average, interspace distance between the fiber surfaces and the coated TiO_2_ layers. These coating structures are confirmed by scanning electron microscopy (SEM) and transmission electron microscopy (TEM). Based on the characterized TiO_2_ coating structures, an energy balance model is developed to describe the light propagation in the TiO_2_-QOFs as functions of the TiO_2_ coating layer structure parameters. From the experiments and model, we find that 91% of the radiant energy delivered to the low patchiness TiO_2_-QOFs propagates on the fiber surfaces as evanescent waves, and the behavior of evanescent waves interacting with the TiO_2_ coating results in a saving of 23% for the radiant energy delivered to the TiO_2_-QOFs. Extending the low patchiness TiO_2_-QOFs from 6.5 to 26 cm is even more beneficial, achieving about 2× carbamazepine degradation and also doubling quantum yields. In addition, the low patchiness coating on the 26 cm fiber uses 77% fewer photocatalysts than the TiO_2_-QOFs with thick and dense TiO_2_ coating layers. Thus, modulating TiO_2_ patchiness on the fiber surface emerges as a tunable parameter to optimize evanescent wave energy rather than refracted light energy to improve both quantum yield and pollutant degradation.

## Results and discussion

### Characterization and analysis of TiO_2_ coating layers

**Fig. 2:**
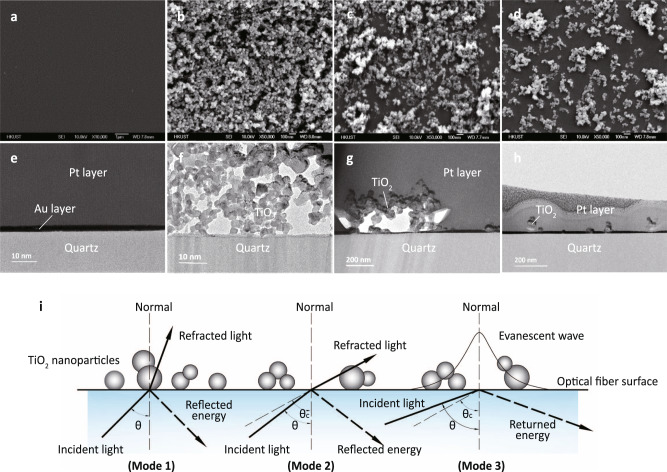
Characterization and analysis of the uncoated quartz optical fibers and three TiO_2_-QOFs. **a**–**d** Scanning electron microscope (SEM) surface images of the uncoated optical fiber, TiO_2_-QOF-High, TiO_2_-QOF-Med, and TiO_2_-QOF-Low, respectively; **e**–**h** transmission electron microscopy (TEM) images of cross-sectional morphologies of the uncoated optical fiber, TiO_2_-QOF-High, TiO_2_-QOF-Med, and TiO_2_-QOF-Low, respectively, in which an Au layer was deposited to capture images during the focus ion beam operation and a Pt layer was deposited to protect the TiO_2_ coating layers from damaging by focus ion beams; and **i** conceptualized images of TiO_2_ coating layers on the quartz optical fiber surfaces and proposed three light propagation modes at the interface.

We fabricated three TiO_2_-QOFs (photos are shown in Supplementary Fig. 3) by dipping quartz optical fibers into P25 suspensions under different conditions^19,20^ (Table 1). They were labeled as TiO_2_-QOF-High, TiO_2_-QOF-Med, and TiO_2_-QOF-Low, which had 81.8, 16.9, and 4.8 μg TiO_2_ per cm^2^ of optical fiber, respectively. Electron microscopy revealed the exterior surface and cross-sectional morphologies of the uncoated optical fiber and the three TiO_2_-QOFs (Fig. 2a–h). As shown in the SEM images (Fig. 2a–d), the exterior surface of the uncoated optical fiber has no TiO_2_ attached (Fig. 2a), while the TiO_2_-QOFs had porous TiO_2_ coating layers. TiO_2_-QOF-High had the densest TiO_2_ coating layer (porosity of 62% (Fig. 2b)). TiO_2_-QOF-Med and TiO_2_-QOF-Low were more porous (71% and 90%, respectively) (Fig. 2c and d). The porous structures of TiO_2_ coating layers were also confirmed by atomic force microscopy (Supplementary Fig. 4). The cross-sections of the uncoated optical fiber and the three TiO_2_-QOFs were prepared by cutting them with a focused ion beam system, and their images (Fig. 2e–h) were obtained by TEM. As shown in Fig. 2e, there was no TiO_2_ attached to the uncoated optical fiber. On TiO_2_-QOF-High (Fig. 2f), TiO_2_ nanoparticles formed a porous TiO_2_ coating layer on the fiber surface. Around 56% of the optical fiber surface had direct contact with TiO_2_ nanoparticles, and interspaces were created between the optical fiber surface and the TiO_2_ coating layers. We define “patchiness” as the ratio (cm^2^/cm^2^) of the optical fiber surface area with direct TiO_2_ contact to the total optical fiber surface area. The calculated patchiness of TiO_2_-QOF-High, TiO_2_-QOF-Med, and TiO_2_-QOF-Low were 56%, 25%, and 8%, respectively (Fig. 2f–h). The decreasing patchiness from TiO_2_-QOF-High to TiO_2_-QOF-Low was also confirmed by a 3D optical profiler (Supplementary Fig. 6).

**Table 1:** Dip-coating conditions and TiO_2_ coating layer parameters in the three TiO_2_-QOFs.

ID	Dip-coating conc.	Dipping duration	Coating/drying cycles	Area-specific TiO_2_ coating density	TiO_2_ coating porosity
	(mg/L)	(min)	/	(μg/cm^2^)	/
TiO_2_-QOF-High	10,000	0.5	5	81.8 ± 5.1	62.2%
TiO_2_-QOF-Med	10,000	0.5	1	16.9 ± 2.5	71.0%
TiO_2_-QOF-Low	40	60	1	4.8 ± 0.6	89.6%

Microscopy also allowed us to conceptualize the porous TiO_2_ coating layers on surfaces of optical fibers as shown in Fig. 2i. The interface is either (i) between the quartz fiber surface and the directly-contact TiO_2_ (termed quartz/TiO_2_ interface) or (ii) between the quartz fiber surface and water diffused from the bulk solution in clusters of TiO_2_ nanoparticles located adjacent to the quartz surface and within the porous coating (termed quartz/water/TiO_2_ interface). Based on the two interfaces, three modes of light propagation are proposed. In Mode 1, as light approaches the quartz/TiO_2_ interface, a large portion of radiant energy refracts into TiO_2_ and a small portion reflects back to quartz due to the higher refractive index of TiO_2_ than quartz. The amount of radiant energy transmitted into TiO_2_ and reflected to quartz is determined by Fresnel equation^26,28^ and is a function of the incident angle (*θ*) of light propagating through the fiber and interacting at the fiber surface, which is the angle between incident light and normal (Fig. 2i). In Mode 2, when light approaches the quartz/water/TiO_2_ interfaces (*θ* < the critical angle of TIR (*θ*_*c*_)), radiant energy is also largely refracted out, and a small portion reflects. In Mode 3, when light approaches the quartz/water/TiO_2_ interfaces (*θ* > *θ*_*c*_), TIR occurs and evanescent waves are generated. Evanescent waves propagate on the surface of optical fibers with a portion being absorbed by TiO_2_. The energy that was not absorbed returns to the optical fibers, making Mode 3 utilize light more efficiently than Modes 1 and 2. To promote Mode 3 of light propagation and generate more evanescent waves, it is desirable to have low TiO_2_ patchiness on the optical fibers. As shown in Fig. 2e–h, TiO_2_-QOF-Low met this requirement.

### Radiant energy dissipation in TiO_2_-QOFs

**Fig. 3:**
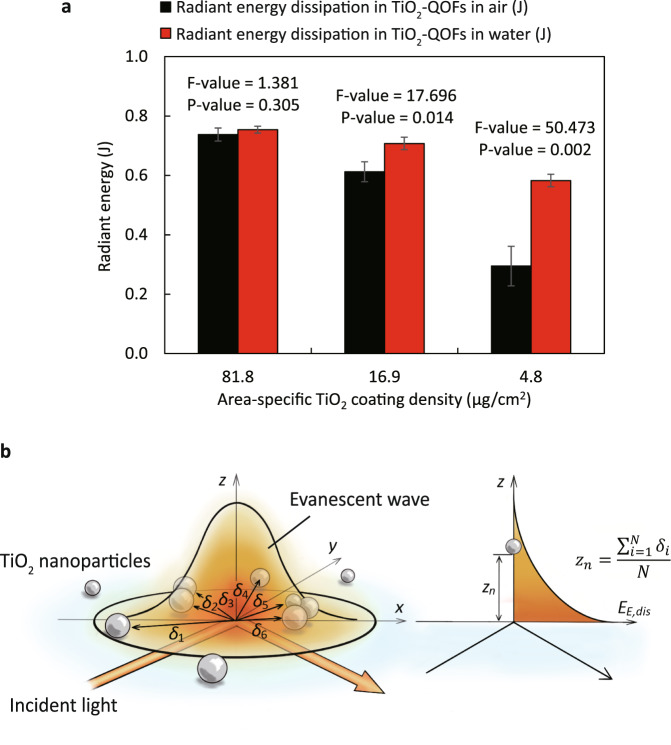
Radiant energy dissipated in TiO_2_-QOFs. **a** Radiant energy dissipated in the three TiO_2_-QOFs when TiO_2_-QOFs were exposed to air and water; **b** schematic of the normalization of the distances between the optical fiber surface and the coated TiO_2_ nanoparticles in an evanescent field (conditions: light intensity = 7.02 mW/cm^2^, wavelength = 365 nm, TiO_2_ coating length = 6.5 cm, irradiation duration = 4 h).

To verify whether evanescent waves existed in our fabricated TiO_2_-QOFs when launching light to the fiber core from an LED, we measured the radiant energy dissipated in TiO_2_-QOFs (*E*_dis_) at different area-specific TiO_2_ coating densities (μg/cm^2^) (Fig. 3a). *E*_dis_ is defined as the radiant energy delivered into but not transmitted through the TiO_2_-QOFs (see “Methods” for details), when TiO_2_-QOFs are exposed to either of two different media (air or water). TiO_2_-QOF-High at 81.8 μg/cm^2^ TiO_2_ showed no statistical difference (*p* > 0.05) in *E*_dis_ between in air and in water. TiO_2_-QOF-Med at 16.9 μg/cm^2^ TiO_2_ and TiO_2_-QOF-Low at 4.8 μg/cm^2^ TiO_2_ showed a higher *E*_dis_ in water than that in air (*p* < 0.05), and the differences were 16% and 98%, respectively.

The higher *E*_dis_ in water was due to the involvement of evanescent waves in TiO_2_-QOFs. Based on the three modes of light propagation illustrated in Fig. 2i, we surmised that *E*_dis_ was caused by the two forms of energy (evanescent waves and refracted light). As the penetration depth of evanescent waves (*Ʌ*) is higher when going from quartz to water than from quartz to air, the radiant energy of evanescent waves dissipated in TiO_2_-QOFs (*E*_E,dis_) varies in either of the two media as shown in Eqs. (1) and (2)^29–31^.

1 $${\Lambda }=\frac{\lambda }{4\pi }{\rm{\cdot }}{\left({{n}_{q}}^{2}{\rm{\cdot }}{{\sin }}^{2}\theta -{{n}_{e}}^{2}\right)}^{-1/2}$$

2 $${E}_{{\mathrm{E}},{{\mathrm{dis}}}}={E}_{i}{\rm{\cdot }}{e}^{-\frac{{z}_{n}}{\Lambda }}$$

where *λ* is the wavelength of light, *n*_*q*_ and *n*_*e*_ are the refractive index of quartz and external medium, respectively, *E*_*i*_ is the radiant energy of incident light, and *z*_*n*_ is the normalized distance between the fiber surface and TiO_2_ nanoparticles at a TIR spot, which is equal to the average distance from the center of an evanescent field to the closest surrounding TiO_2_ nanoparticles within the evanescent field (*δ*_*i*_), as shown in Fig. 3b^32,33^. In our study, the LED lamp source was fixed at one end of a quartz optical fiber, so *θ* remained the same and was always between 0.376π and 0.495π (calculation shown in Supplementary Note 5A). *E*_*i*_ and *λ* are inherent properties of the LED, *n*_*q*_ is an inherent property of quartz optical fibers, and *z*_*n*_ is a structural property of the TiO_2_ coating layer. As such, these values did not change during the experiments. The *n*_*e*_ of water is higher than the *n*_*e*_ of air (1.33 versus 1.00), so evanescent waves penetrate further from quartz to water than to air suggested by Eq. (1). Therefore, *E*_E,dis_ in water is higher than *E*_E,dis_ in air as given by Eq. (2).

Unlike in evanescence wave energy, the radiant energy of refracted light dissipated in TiO_2_-QOFs (*E*_R,dis_) is constant in the two media. At the quartz/air or quartz/water interface, *θ* was always greater than *θ*_c_ of TIR, which suggests no mode 2 of light propagation and no radiant energy refracted from quartz to air or from quartz to water. *E*_R,dis_ only occurred on the quartz/TiO_2_ interface, calculated using Fresnel equation shown in Eq. (3):

3 $${E}_{{\mathrm{R}},{{\mathrm{dis}}}}={E}_{i}{\rm{\cdot }}\left\{1-\frac{1}{2}\left\{{\left[\frac{{n}_{q}{\cos }\theta -{n}_{T}\sqrt{1-{\left(\frac{{n}_{q}}{{n}_{T}}{\sin }\theta \right)}^{2}}}{{n}_{q}{\cos }\theta +{n}_{T}\sqrt{1-{\left(\frac{{n}_{q}}{{n}_{T}}{\sin }\theta \right)}^{2}}}\right]}^{2}+{\left[\frac{{n}_{q}\sqrt{1-{\left(\frac{{n}_{q}}{{n}_{T}}{\sin }\theta \right)}^{2}}-{n}_{T}{\cos }\theta }{{n}_{q}\sqrt{1-{\left(\frac{{n}_{q}}{{n}_{T}}{\sin }\theta \right)}^{2}}+{n}_{T}{\cos }\theta }\right]}^{2}\right\}\right\}$$

where *n*_*q*_ and *n*_*T*_ are refractive indexes of quartz and TiO_2_, respectively. As demonstrated in Eq. (3), *E*_*i*_ and *θ* remained the same in the two media as mentioned before. *n*_*q*_ and *n*_*T*_ are inherent properties of quartz and TiO_2_, respectively, which also did not change. Thus, *E*_R,dis_ remains constant regardless of the external medium being air or water. Therefore, the variable nature of *E*_E,dis_ and constant feature of *E*_R,dis_ cause differences in *E*_dis_ and thus verify the existence of evanescent waves in TiO_2_-QOFs. The existence of evanescent waves was also proven by tracking the irradiance loss in a UV irradiated uncoated fiber immersed in methylene blue solutions (Supplementary Note 5B).

### Modeling light propagation in TiO_2_-QOFs

**Fig. 4:**
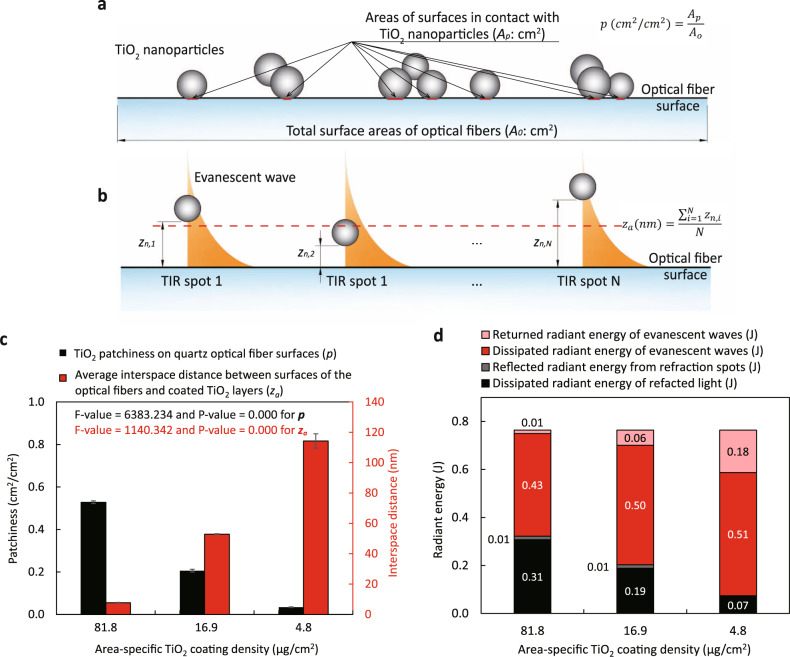
Modeling results of light propagation in the three TiO_2_-QOFs. **a** Schematic showing TiO_2_ patchiness on quartz optical fibers (*p*); **b** schematic showing average interspace distance between fiber surfaces and TiO_2_ coating layers (*z*_*a*_); **c** TiO_2_ coating layer structure parameters (*p* and *z*_*a*_) of the three TiO_2_-QOFs; and **d** modeling results of the dissipated radiant energy of evanescent waves (${E}_{{\mathrm{E}},{{\mathrm{dis}}}}{\prime}$) and refracted light (${E}_{{\mathrm{R}},{{\mathrm{dis}}}}{\prime}$) as well as the returned radiant energy of evanescent waves (*E*_E,return_) and the reflected radiant energy from refraction spots (*E*_R,reflect_) in the three TiO_2_-QOFs (conditions: light intensity = 7.02 mW/cm^2^, wavelength = 365 nm, TiO_2_ coating length = 6.5 cm, irradiation duration = 4 h).

We developed an energy balance model to simulate the light propagation along TiO_2_-QOFs using geometrical optics in accordance with our schematics of TiO_2_ coating layers on the quartz optical fibers and the 3 modes of light propagation. In this model, light rays at different angles are emitted from an LED light source and launched into the TiO_2_-QOFs. After entering TiO_2_-QOFs, each ray strikes the inner surface of TiO_2_-QOFs with *θ* between 0.376π and 0.495π. The model assumes *θ* is discrete with an increment of 0.0001π, and each light ray has the same amount of radiant energy (*E*_0_). Each light ray continuously strikes the inner TiO_2_-QOF surfaces, which (i) generates evanescent waves at the quartz/water/TiO_2_ interface and activates TiO_2_ and/or returns into the fiber, or (ii) activates attached TiO_2_ through refraction at the quartz/TiO_2_ interface with a small portion reflected to the fiber. Based on the above assumptions and Eqs. (1–3), the dissipated radiant energy of evanescent waves $({E}_{{\mathrm{E}},{{\mathrm{dis}}}}{\prime})$ and that of refracted light $({E}_{{\mathrm{R}},{{\mathrm{dis}}}}{\prime})$ of each light ray are represented by the summation of radiant energy dissipated at each TIR spot and refraction spot along TiO_2_-QOFs, respectively. The deterministic form of the equations is shown in Eqs. (4–6) (see Supplementary Note 5C for detailed derivation).

4 $${E}_{{\mathrm{E}},{{\mathrm{dis}}}}{\prime} ={E}_{0}{\rm{\cdot }}\frac{\left(1-p\right){\rm{\cdot }}{e}^{-\frac{{z}_{a}}{\Lambda }}{\rm{\cdot }}\left\{1-{\left[\left(1-p\right){\rm{\cdot }}\left(1-{e}^{-\frac{{z}_{a}}{\Lambda }}\right)+p{\rm{\cdot }}\left(1-T\right)\right]}^{L/(d{\rm{\cdot }}{\tan }\theta )}\right\}}{\left(1-p\right)\cdot {e}^{-\frac{{z}_{a}}{\Lambda }}+p\cdot T}$$

5 $${E}_{{\mathrm{R}},{{\mathrm{dis}}}}{\prime} ={E}_{0}{\rm{\cdot }}\frac{p{\rm{\cdot }}T{\rm{\cdot }}\left\{1-{\left[(1-p){\rm{\cdot }}\left(1-{e}^{-\frac{{z}_{a}}{\Lambda }}\right)+p{\rm{\cdot }}\left(1-T\right)\right]}^{L/(d{\rm{\cdot }}{\tan }\theta )}\right\}}{(1-p){\rm{\cdot }}{e}^{-\frac{{z}_{a}}{\Lambda }}+p{\rm{\cdot }}T}$$

where *p* is the TiO_2_ patchiness on quartz optical fibers (Fig. 4a), *z*_*a*_ is the average interspace distance between the optical fiber surface and the coated TiO_2_ layer (nm), which equals to the average value of *z*_*n*_ at all TIR spots along the fiber length (Fig. 4b), *L* is the TiO_2_ coating length (cm), and *d* is the diameter of optical fibers (cm). *E*_dis_ equals to the summation of ${E}_{{\mathrm{E}},{{\mathrm{dis}}}}{\prime}$ and ${E}_{{\mathrm{R}},{{\mathrm{dis}}}}{\prime}$ at all incident rays with *θ* from 0.376π to 0.495π as shown in Eq. (6).

6 $${E}_{{{\mathrm{dis}}}}=\mathop{\sum }\limits_{\theta =0.376\pi }^{0.495\pi }{E}_{{\mathrm{E}},{{\mathrm{dis}}}}{\prime} +\mathop{\sum }\limits_{\theta =0.376\pi }^{0.495\pi }{E}_{{\mathrm{R}},{{\mathrm{dis}}}}{\prime}$$

For our aqueous pollutant degradation study, an LED was attached to a single quartz optical fiber centered in the axial (i.e., longitudinal direction) of a tubular reactor filled with water containing carbamazepine. *E*_dis_ is a function of *E*_0_, *L*, *d*, and *n*_*e*_, while *p* and *z*_*a*_ are intrinsic properties of the TiO_2_ coating layer and relate only to the coating structure but not the experimental conditions. *p* and *z*_*a*_ for each TiO_2_-QOF can then be calculated by substituting *E*_0_, *L*, *d*, *n*_*e*_, and *E*_dis_ in air or water obtained by optical measurements when a TiO_2_-QOF is surrounded by air or immersed in water into Eqs. (4–6). Three experimental conditions were used to calculate the average *p* and *z*_*a*_ of each TiO_2_-QOF (detailed calculations for each condition shown in Supplementary Note 5D). The results showed *p* and *z*_*a*_ under the three conditions varied by <5% (Supplementary Table 2). The model’s accuracy was also validated by comparing the measured *E*_dis_ with the model-predicted values for incident light irradiance from 0.41 to 7.02 mW/cm^2^ (details in Supplementary Note 5E).

The average *p* and *z*_*a*_ for TiO_2_-QOF-High were 0.528 cm^2^/cm^2^ and 7.7 nm, respectively. For TiO_2_-QOF-Med, *p* and *z*_*a*_ were 0.206 cm^2^/cm^2^ and 52.9 nm, respectively, and for TiO_2_-QOF-Low, they were 0.034 cm^2^/cm^2^ and 114.3 nm, respectively (shown in Fig. 4c). These results were consistent with the TiO_2_ coating structure parameters we observed in the TEM images shown in Fig. 2. Within the same TiO_2_-QOF, similar *p* and *z*_*a*_ calculated from the model under the three conditions further confirmed they only related to the coating structures rather than to experimental conditions.

Based on the average *p* and *z*_*a*_ obtained above, ${E}_{{\mathrm{E}},{{\mathrm{dis}}}}{\prime}$ and ${E}_{{\mathrm{R}},{{\mathrm{dis}}}}{\prime}$ can be calculated using Eqs. (4) and (5). In addition, to close the energy balance, the returned radiant energy of evanescent waves (*E*_E,return_) and the reflected radiant energy from refraction spots (*E*_R,reflect_) were calculated using Eqs. (7) and (8) (see Supplementary Note 5F for derivation).

7 $${E}_{{\mathrm{E}},{{\mathrm{return}}}}=\mathop{\sum }\limits_{\theta =0.376\pi }^{0.495\pi }\left({E}_{0}-{E}_{{\mathrm{E}},{{\mathrm{dis}}}}{\prime} -{E}_{{\mathrm{R}},{{\mathrm{dis}}}}{\prime} \right){\rm{\cdot }}\frac{(1-p){\rm{\cdot }}\left(1-{e}^{-\frac{{z}_{a}}{\Lambda }}\right)}{p{\rm{\cdot }}\left(1-T\right)+(1-p){\rm{\cdot }}\left(1-{e}^{-\frac{{z}_{a}}{\Lambda }}\right)}$$

8 $${E}_{{\mathrm{R}},{{\mathrm{reflect}}}}=\mathop{\sum }\limits_{\theta =0.376\pi }^{0.495\pi }\left({E}_{0}-{E}_{{\mathrm{E}},{{\mathrm{dis}}}}{\prime} -{E}_{{\mathrm{R}},{{\mathrm{dis}}}}{\prime} \right){\rm{\cdot }}\frac{p{\rm{\cdot }}\left(1-T\right)}{p{\rm{\cdot }}\left(1-T\right)+(1-p){\rm{\cdot }}\left(1-{e}^{-\frac{{z}_{a}}{\Lambda }}\right)}$$

Fig. 4d compares the calculated ${E}_{{\mathrm{E}},{{\mathrm{dis}}}}{\prime}$, ${E}_{{\mathrm{R}},{{\mathrm{dis}}}}{\prime}$, *E*_E,return_, and *E*_R,reflect_ in water against area-specific TiO_2_ coating densities. As the area-specific TiO_2_ coating density decreased from TiO_2_-QOF-High to TiO_2_-QOF-Med to TiO_2_-QOF-Low, the generated evanescent wave energy (*E*_E,g_)—i.e., the sum of ${E}_{{\mathrm{E}},{{\mathrm{dis}}}}{\prime}$ and *E*_E,return_,—increased from 0.44 to 0.56 to 0.69J, and the generated refracted light energy (*E*_R,g_)—i.e., the sum of ${E}_{{\mathrm{R}},{{\mathrm{dis}}}}{\prime}$ and *E*_R,reflect_,—decreased from 0.32 to 0.20 to 0.07J. The increasing *E*_E,g_ and decreasing *E*_R,g_ from TiO_2_-QOF-High to TiO_2_-QOF-Med and then to TiO_2_-QOF-Low confirmed that TiO_2_-QOF-Low, which has the lowest TiO_2_ patchiness, promotes Mode 3 and generates more evanescent waves.

We further compared the ratios of ${E}_{{\mathrm{R}},{{\mathrm{dis}}}}{\prime}$ to *E*_R,g_ and those of ${E}_{{\mathrm{E}},{{\mathrm{dis}}}}{\prime}$ to *E*_E,g_ in the three TiO_2_-QOFs. All ratios of ${E}_{{\mathrm{R}},{{\mathrm{dis}}}}{\prime}$ to *E*_R,g_ were close to 1, confirming that refracted light mostly propagated away from TiO_2_-QOFs and its radiant energy dissipated out. However, with increasing *E*_E,g_ from TiO_2_-QOF-High to TiO_2_-QOF-Low, the ratios of ${E}_{{\mathrm{E}},{{\mathrm{dis}}}}{\prime}$ to *E*_E,g_ decreased from 0.98 to 0.74. This is because *z*_*a*_ of TiO_2_-QOFs modulates the evanescent wave energy returning to optical fibers. The value of *z*_*a*_ in TiO_2_-QOF-High (7.7 nm) is much smaller than the value of *Λ* from quartz to water (50–120 nm) (Supplementary Fig. 11), suggesting evanescent wave energy dissipates when they reach the closest TiO_2_ nanoparticles and a negligible amount of them returned to the optical fibers to give a highest ratio of ${E}_{{\mathrm{E}},{{\mathrm{dis}}}}{\prime}$ to *E*_E,g_. As increasing *z*_*a*_ from 7.7 nm in TiO_2_-QOF-High to 52.9 nm in TiO_2_-QOF-Med and 114.3 nm in TiO_2_-QOF-Low, more evanescent waves returned to the optical fibers (Supplementary Fig. 12) because they cannot reach the TiO_2_ coating layers to give lower ratios of ${E}_{{\mathrm{E}},{{\mathrm{dis}}}}{\prime}$ to *E*_E,g_. Those trends were also observed when the TiO_2_-QOFs were exposed to air (Supplementary Fig. 13). The above modeling results suggest the TiO_2_ layer structure parameters (*p* and *z*_*a*_) are important to control the light energy within TiO_2_-QOFs, while the TiO_2_ coating thickness is not critical.

### Degradation of carbamazepine by TiO_2_-QOFs

**Fig. 5:**
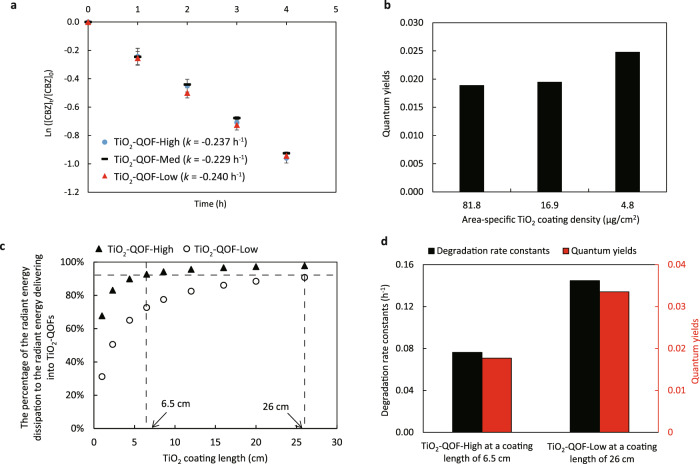
Carbamazepine degradation. **a** Pseudo-first order degradation kinetics; **b** quantum yields of carbamazepine degradation by the three TiO_2_-QOFs; **c** the percentage of the radiant energy dissipation to the radiant energy delivered to TiO_2_-QOFs as a function of TiO_2_ coating length; and **d** comparison between degradation rate constants and apparent quantum yields by the UV irradiated TiO_2_-QOF-High at a coating length of 6.5 cm and TiO_2_-QOF-Low at a coating length of 26 cm in a 70 mL reactor (conditions: light intensity = 7.02 mW/cm^2^, light wavelength = 365 nm, [CBZ]_0_ = 2 μM, irradiation duration = 4 h).

The above data, models, and insights were based on light measurements outside and along the length of fibers coated with TiO_2_. We then examined the impact of increasing energy efficiency of the low patchiness TiO_2_ coating layer to its photocatalytic performance in carbamazepine degradation. Control tests confirmed that carbamazepine was not adsorbed by TiO_2_-QOFs in the dark or not degraded when light was launched from LEDs into an uncoated optical fiber (Supplementary Figs. 14a and b). Even though the radiant energy dissipation varied in the three TiO_2_-QOFs, launching 365 nm light from an LED separately into the three TiO_2_-QOFs resulted in statistically the same (*p* = 0.450) pseudo-first-order degradation kinetics for carbamazepine in water (rate constants *k*, shown in Fig. 5a). The calculated quantum yields of carbamazepine degradation (i.e., moles of carbamazepine degraded per mole of photons absorbed by TiO_2_ coating layers) increased from 0.0189 in TiO_2_-QOF-High to 0.0248 in TiO_2_-QOF-Low (Fig. 5b). The improved quantum yield was attributed to the highest quantity of evanescent waves generated in TiO_2_-QOF-Low saving more radiant energy than the other two. The evanescent waves generated in TiO_2_-QOF-Low allow light to be evenly dissipated along the fiber, which prevents local photon oversaturation at the beginning sections of TiO_2_-QOFs and thus reduces its associated efficiency losses (Supplementary Note 6A). We also proved that the improved quantum yield was not attributed to the mass transfer limitation (Supplementary Note 6B) or carbamazepine adsorption (Supplementary Fig. 14c) of different TiO_2_ coating layers. We then fabricated two new TiO_2_-QOFs, i.e., TiO_2_-QOF-Low″ at *p* of 0.018 and *z*_*a*_ of 139.50 nm and TiO_2_-QOF-Low′ at *p* of 0.026 and *z*_*a*_ of 127.97 nm. Both degradation rate constants and quantum yields decreased with decreasing *p* and increasing *z*_*a*_ (Supplementary Table 5).

Quantum yields of carbamazepine degradation by the TiO_2_-QOF-Low are 1–50× higher than those in the UV-based advanced oxidation processes^34–36^ (Supplementary Table 6). The quantum yield of TiO_2_-QOF-Low can be further increased by extending its length to fully utilize the returned radiant energy of evanescent waves in TiO_2_-QOF-Low and to increase its photocatalytic reactive sites. Figure 5c shows the percentage of radiant energy dissipation relative to total radiant energy delivered into TiO_2_-QOFs with different coatings. Denser coatings on TiO_2_-QOF-High resulted in >92% of the energy launched into the fiber being dissipated within the first 6.5 cm of the coated length, whereas low patchiness TiO_2_-QOF-Low utilized the same amount light over 26 cm of the coated length. The 4× increase in length also increased by 4× the surface areas available for ROS production that degrades pollutants, therefore, the 26-cm TiO_2_-QOF-Low was estimated to achieve 2× degradation rate constants and 2× apparent quantum yields (moles of carbamazepine degraded per moles of photons launched to optical fibers) compared with TiO_2_-QOF-High at a coating length of 6.5 cm (Supplementary Note 6D). The higher rate constant and apparent quantum yield of 26-cm TiO_2_-QOF-Low was further experimentally confirmed in a 70 mL reactor (Fig. 5d). Even if refracted light emitted from TiO_2_-QOF-High can be utilized by bundling TiO_2_-QOFs together^37^, optimizing evanescent waves to fully use transmitted light in longer TiO_2_-QOF-Low is 44–96% more efficient (in carbamazepine degradation rates and apparent quantum yields) than harvesting refracted light by using TiO_2_-QOF-High bundles (detailed see Supplementary Note 6E). By controlling surface patchiness and distance between fiber surface and photocatalyst coating layers, TiO_2_-QOF-Low not only prevent light oversaturation and its associated efficient losses, but also reduces light wasted by refraction and increase surface reactive sites. These features make TiO_2_-QOF-Low more energy-efficient to degrade pollutants. In addition, the 26-cm TiO_2_-QOF-Low used 77% fewer TiO_2_ than the 6.5-cm TiO_2_-QOF-High, although the former was 4× longer, and it had more surface areas available for photocatalytic reactions. The TiO_2_ coating in TiO_2_-QOF-Low is also stable, and there was no TiO_2_ coming off during the reaction (Supplementary Fig. 19).

### Implications for photocatalytic reactor design

Photocatalytic processes have broad application in water and air treatment, energy production, organic synthesis, and other fields. Less than 1 in 100 papers address the critical barrier to making photocatalytic reactors more effective, namely light and energy management; the other papers focus largely on discovery of new or incremental improvement in existing photocatalyst materials^10^. An important but often overlooked aspect to limiting use of photocatalytic processes into engineering practice is the influence of photocatalytic reactor design, rather than material properties alone. To address the barrier of reactor design rather than material properties we show how quantum yields are effectively increased more significantly by managing the way light reaches the catalyst, than recent improvements in catalyst materials themselves. In addition to considering only refracted light in our optical fiber reactor, another key insight was the separation of photocatalyst activation by refracted light versus evanescent wave energy to activate the photocatalyst. We report for the first time here the relative importance of both mechanisms, as well as how to modulate their relative importance. Here, we developed a novel coating strategy that left 3.4% of the optical fiber surface in direct contact with TiO_2_ and, on-average, 114.3 nm interspace distance between the fiber surface and the coated TiO_2_ layers. This strategy successfully reduced refraction, generated evanescent waves in the TiO_2_-QOF, and thus allowed even dissipation of light along TiO_2_-QOF to prevent oversaturation of the light delivered to the fiber and its associated efficiency losses. The novel TiO_2_-QOF enables the design of a photocatalytic reactor that uses 77% less mass of photocatalysts but achieves up to 96% improvement in quantum yields compared with reactors built of fibers with densely coated TiO_2_ layers, mainly because optimizing evanescent waves to fully use transmitted light in longer TiO_2_-QOF-Low is more efficient than harvesting refracted light by using TiO_2_-QOF-High bundles. Moreover, such a strategy of managing evanescent wave energy is applicable to all photocatalysts and thus emerges as an important cost consideration especially when contemplating expensive photocatalysts.

A new platform can also be established using novel photocatalyst-coated optical fibers to allow evanescent wave energy to activate photocatalysts. This new platform can quantify performance of photocatalysts more precisely by minimizing the radiation scattering by photocatalysts and water parameters, preventing aggregation of photocatalysts to maximize the interaction between reactive sites and target compounds, and allowing easier and more accurate quantification of photons absorbed by photocatalysts.

## Methods

### Coating quartz optical fibers with TiO_2_ layers

Uncoated optical fibers (FT1000UMT, Thorlabs) were prepared by cutting the fibers into segments of specified lengths, stripping the buffer coating and cladding, and polishing both tips (described in Supplementary Method A). TiO_2_ suspension was prepared by dispersing TiO_2_ (P25) in double deionized water (18.2 MΩ-cm). TiO_2_-QOFs were fabricated by dipping the optical fiber segments in TiO_2_ suspension at different conditions (Table 1) to produce TiO_2_ layers with different area-specific TiO_2_ coating densities^20^. TiO_2_-QOF-High was fabricated by completing 5 cycles of dipping the segments in a 10,000 mg/L TiO_2_ suspension for 0.5 min and air drying for another 0.5 min. TiO_2_-QOF-Med was fabricated following a similar method, but with only one dip-coating/drying cycle. TiO_2_-QOF-Low was fabricated by dipping the segments in a 40 mg/L TiO_2_ suspension for 1 h followed by air drying.

### Characterization of TiO_2_-QOFs

The images of coated and uncoated fiber surfaces were obtained by a SEM (JSM-6700F, JEOL) and an AFM (Dimension 3100, Digital Instruments). The cross-sections were prepared by a FIB following a standard preparation method^38^ (details in Supplementary Method B), before characterization by a TEM (JEM-100CXII, JEOL). The cross-sections were also characterized and confirmed by a 3D surface optical profilometer (Wyko NT 3300, Veeco). The TiO_2_ layer masses (*m*_TiO2_) on the optical fibers were measured gravimetrically by the weight of the optical fibers before and after the dip-coating/drying cycles. The porosity of the TiO_2_ coating layers, which is defined as the fraction of the total pore volume over the volume of the TiO_2_ layer, was calculated using Eq. (9).

9 $${{\mathrm{Porosity}}}=1-\frac{{m}_{{{\mathrm{TiO}}}2}}{{\rho }_{{{\mathrm{TiO}}}2}{\rm{\cdot }}L{\rm{\cdot }}\pi d{\rm{\cdot }}D}$$

where *ρ*_TiO2_ is the true density of the TiO_2_ particles (4.26 g/mL at 25 °C obtained from Sigma-Aldrich), *L* is the TiO_2_ coating length, *d* is the diameter of optical fibers, and *D* is the thickness of the TiO_2_ coating layers, which was determined by the cross-sectional profiles of TiO_2_-QOFs from SEM images. The TiO_2_ patchiness was calculated by dividing the optical fiber surface in direct contact with TiO_2_ nanoparticles by the total surface of the optical fiber determining from TEM cross-section images.

### Photocatalytic experiment

The photocatalytic experiment including irradiance measurement and carbamazepine degradation was conducted in a mixed batch reactor as shown in Supplementary Fig. 7. The reactor was composed of a cylindrical glass vessel (23 mL) with a length of 65 mm and an inner diameter of 25 mm, a magnetic stirrer (F203A0160, VELP) at the bottom for rapid mixing, a 365 nm LED light source (H44LV1C0, HPLighting), and an optical meter (RPS900-R, International Light Technologies). A single TiO_2_-QOF was fixed in the reactor with one end mounted to the LED and the other end connected to the optical meter to obtain the transmitted irradiance after the 6.5 cm light path. All the experiments were conducted in triplicate, and TiO_2_-QOFs were fabricated using the same preparation method in every repeated experiment. The radiant energy dissipated in the TiO_2_-QOFs (*E*_dis_: J) was expressed as the difference between radiant energy delivered into TiO_2_-QOFs (*E*_in_) and the radiant energy transmitted to the terminal end (*E*_out_). The radiant energy of the uncoated optical fiber measured at the terminal end in air was regarded as *E*_in_, because light delivered into an uncoated optical fiber is totally reflected, and attenuation along the fiber is negligible in a very short propagation distance. *E*_dis_ is expressed as Eq. (10).

10 $${E}_{{{\mathrm{dis}}}}=\left({I}_{{{\mathrm{in}}}}-{I}_{{{\mathrm{out}}}}\right){\rm{\cdot }}A{\rm{\cdot }}t$$

where *I*_*i*_ is the irradiance measured from the optical meter (W/cm^2^), *A* is the cross-sectional area of optical fibers (cm^2^), and *t* is the irradiation duration (s). Carbamazepine concentrations were determined using a high-performance liquid chromatograph (VP series, Shimadzu) equipped with a Waters symmetry C18 column and a UV-Vis detector. The degradation kinetic was obtained by plotting carbamazepine concentration as a function of time to obtain the reaction order and rate constants (*k*: h^−1^). The quantum yields (*η*) of the degradation was calculated using Eq. (11).

11 $$\eta =\frac{k{\rm{\cdot }}V{\rm{\cdot }}{\left({\left[{CBZ}\right]}_{0}\right)}^{1}}{{I}_{{dis}}{\rm{\cdot }}A}$$

where [*CBZ*]_0_ is the initial carbamazepine concentration (mole/L), *V* is the liquid volume (L), and *I*_dis_ is the irradiance dissipated in the TiO_2_-QOFs (mol-photons/(cm^2^·h)).

## Supplementary information

Supplementary Information

Peer Review File

## Data Availability

The authors declare that the data that support the findings of this study are available from the corresponding author upon reasonable request.

## References

[1] Herrmann JM (1999). Heterogeneous photocatalysis: fundamentals and applications to the removal of various types of aqueous pollutants. Catal. Today.

[2] Yin R (2020). Degradation of aliphatic halogenated contaminants in water by UVA/Cu–TiO_2_ and UVA/TiO_2_ photocatalytic processes: Structure-activity relationship and role of reactive species. Chemosphere.

[3] Carbonaro S, Sugihara MN, Strathmann TJ (2013). Continuous-flow photocatalytic treatment of pharmaceutical micropollutants: Activity, inhibition, and deactivation of TiO_2_ photocatalysts in wastewater effluent. Appl. Catal. B.

[4] Dalrymple OK, Stefanakos E, Trotz MA, Goswami DY (2010). A review of the mechanisms and modeling of photocatalytic disinfection. Appl. Catal. B.

[5] Kudo A, Miseki Y (2009). Heterogeneous photocatalyst materials for water splitting. Chem. Soc. Rev..

[6] Lang X, Chen X, Zhao J (2014). Heterogeneous visible light photocatalysis for selective organic transformations. Chem. Soc. Rev..

[7] Fujishima A, Rao TN, Tryk DA (2000). Titanium dioxide photocatalysis. J. Photochem. Photobiol. C..

[8] Doll TE, Frimmel FH (2005). Photocatalytic degradation of carbamazepine, clofibric acid and iomeprol with P25 and Hombikat UV100 in the presence of natural organic matter (NOM) and other organic water constituents. Water Res..

[9] Gaya UI, Abdullah AH (2008). Heterogeneous photocatalytic degradation of organic contaminants over titanium dioxide: A review of fundamentals, progress and problems. J. Photochem. Photobiol. C..

[10] Loeb SK (2019). The technology horizon for photocatalytic water treatment: Sunrise or sunset?. Environ. Sci. Technol..

[11] Satuf ML, Brandi RJ, Cassano AE, Alfano OM (2007). Quantum efficiencies of 4-chlorophenol photocatalytic degradation and mineralization in a well-mixed slurry reactor. Ind. Eng. Chem. Res..

[12] Curcó D, Giménez J, Addardak A, Cervera-March S, Esplugas S (2002). Effects of radiation absorption and catalyst concentration on the photocatalytic degradation of pollutants. Catal. Today.

[13] Blatchley ER (2008). Validation of large-scale, monochromatic UV disinfection systems for drinking water using dyed microspheres. Water Res..

[14] Marinangeli RE, Ollis DF (1977). Photoassisted heterogeneous catalysis with optical fibers: I. Isolated single fiber. AIChE J..

[15] Marinangeli RE, Ollis DF (1980). Photo-assisted heterogeneous catalysis with optical fibers II. Nonisothermal single fiber and fiber bundle. AIChE J..

[16] Hofstadler K, Bauer R, Novallc S, Heisler G (1994). New reactor design for photocatalytic wastewater treatment with TiO_2_ immobilized on fused-silica glass fibers: photomineralization of 4-chlorophenol. Environ. Sci. Technol..

[17] Peill NJ, Hoffmann MR (1995). Development and optimization of a TiO_2_-coated fiber-optic cable reactor: photocatalytic degradation of 4-chlorophenol. Environ. Sci. Technol..

[18] Zhang S (2012). Design of H_3_PW_12_O_40_/TiO_2_ and Ag/H_3_PW_12_O_40_/TiO_2_ film-coated optical fiber photoreactor for the degradation of aqueous rhodamine B and 4-nitrophenol under simulated sunlight irradiation. Chem. Eng. J..

[19] Choi W, Ko JY, Park H, Chung JS (2001). Investigation on TiO_2_-coated optical fibers for gas-phase photocatalytic oxidation of acetone. Appl. Catal. B.

[20] Ling L (2017). Coupling light emitting diodes with photocatalyst-coated optical fibers improves quantum yield of pollutant oxidation. Environ. Sci. Technol..

[21] Danion A, Disdier J, Guillard C, Jaffrezic-Renault N (2007). Malic acid photocatalytic degradation using a TiO_2_-coated optical fiber reactor. J. Photochem. Photobiol. A.

[22] Peill NJ, Hoffmann MR (1996). Chemical and physical characterization of a TiO_2_-coated fiber optic cable reactor. Environ. Sci. Technol..

[23] Peill NJ, Hoffmann MR (1998). Mathematical model of a photocatalytic fiber-optic cable reactor for heterogeneous photocatalysis. Environ. Sci. Technol..

[24] Chen D, Li F, Ray AK (2000). Effect of mass transfer and catalyst layer thickness on photocatalytic reaction. AIChE J..

[25] Chen Y (2015). Study on the propagation mechanism of evanescent waves in one-dimensional periodic photonic crystal. Phys. Lett. A.

[26] Peatross, J. & Michael, W. *Physics of Light and Optics* (Brigham Young Univ. Press, 2017).

[27] Essiambre, R. J., Tkach, R. W. & Ryf, R. in *Optical Fiber Telecommunications VIB: Systems and Networks: Sixth Edition* 1–43 (Academic Press, 2013).

[28] Hui, R. & O’Sullivan, M. in *Fiber Optic Measurement Techniques* 1–128 (Academic Press, 2009).

[29] Axelrod D (2001). Total internal reflection fluorescence microscopy in cell biology. Traffic.

[30] Sarkar A, Robertson RB, Fernandez JM (2004). Simultaneous atomic force microscope and fluorescence measurements of protein unfolding using a calibrated evanescent wave. Proc. Natl Acad. Sci. USA.

[31] Lensun L, Smith TA, Gee ML (2002). Partial denaturation of silica-adsorbed bovine serum albumin determined by time-resolved evanescent wave-induced fluorescence spectroscopy. Langmuir.

[32] Cragg GE, So PT (2000). Lateral resolution enhancement with standing evanescent waves. Opt. Lett..

[33] Zhu L (2015). Metal-dielectric waveguides for high efficiency fluorescence imaging. J. Phys. Chem. C..

[34] Sun J (2019). The influence of the UV/chlorine advanced oxidation of natural organic matter for micropollutant degradation on the formation of DBPs and toxicity during post-chlorination. Chem. Eng. J..

[35] Pan Y (2017). UV/chlorine treatment of carbamazepine: Transformation products and their formation kinetics. Water Res..

[36] Keen OS, Baik S, Linden KG, Aga DS, Love NG (2012). Enhanced biodegradation of carbamazepine after UV/H_2_O_2_ advanced oxidation. Environ. Sci. Technol..

[37] O’Neal Tugaoen H, Garcia-Segura S, Hristovski K, Westerhoff P (2018). Compact light-emitting diode optical fiber immobilized TiO_2_ reactor for photocatalytic water treatment. Sci. Total Environ..

[38] Sezen, M. in *Modern Electron Microscopy in Physical and Life Sciences* (IntechOpen Press, 2016).

