# Supplementary material for: Evanescent waves modulate energy efficiency of photocatalysis within TiO2 coated optical fibers illuminated using LEDs
Source: Nat Commun. 2021 Jul 2;12:4101. doi: 10.1038/s41467-021-24370-8 (PMC8253814; doi:10.1038/s41467-021-24370-8)
Supplement: Supplementary file 1 — Supplementary Information [file 41467_2021_24370_MOESM1_ESM.pdf]

Supplementary Information

Evanescent waves modulate energy efficiency of photocatalysis within TiO_2 coated optical fibers illuminated using LEDs

Submitted to:

Nature Communications

Yinghao Song[1], Li Ling[1], Paul Westerhoff[2], Chii Shang[1,3]

1. Department of Civil and Environmental Engineering, Hong Kong University of Science and Technology, Hong Kong, China

2. School of Sustainable Engineering and the Built Environment and Nanosystems Engineering Research Center for Nanotechnology-Enabled Water Treatment (NEWT), Arizona State University, Box 3005, Tempe, Arizona 85287-3005, United States

3. Hong Kong Branch of Chinese National Engineering Research Center for Control & Treatment of Heavy Metal Pollution, the Hong Kong University of Science and Technology, Clear Water Bay, Kowloon, Hong Kong, China

*These authors jointly supervised this work: Li Ling, e-mail: celingli@ust.hk; and Chii Shang, e-mail: cechii@ust.hk.

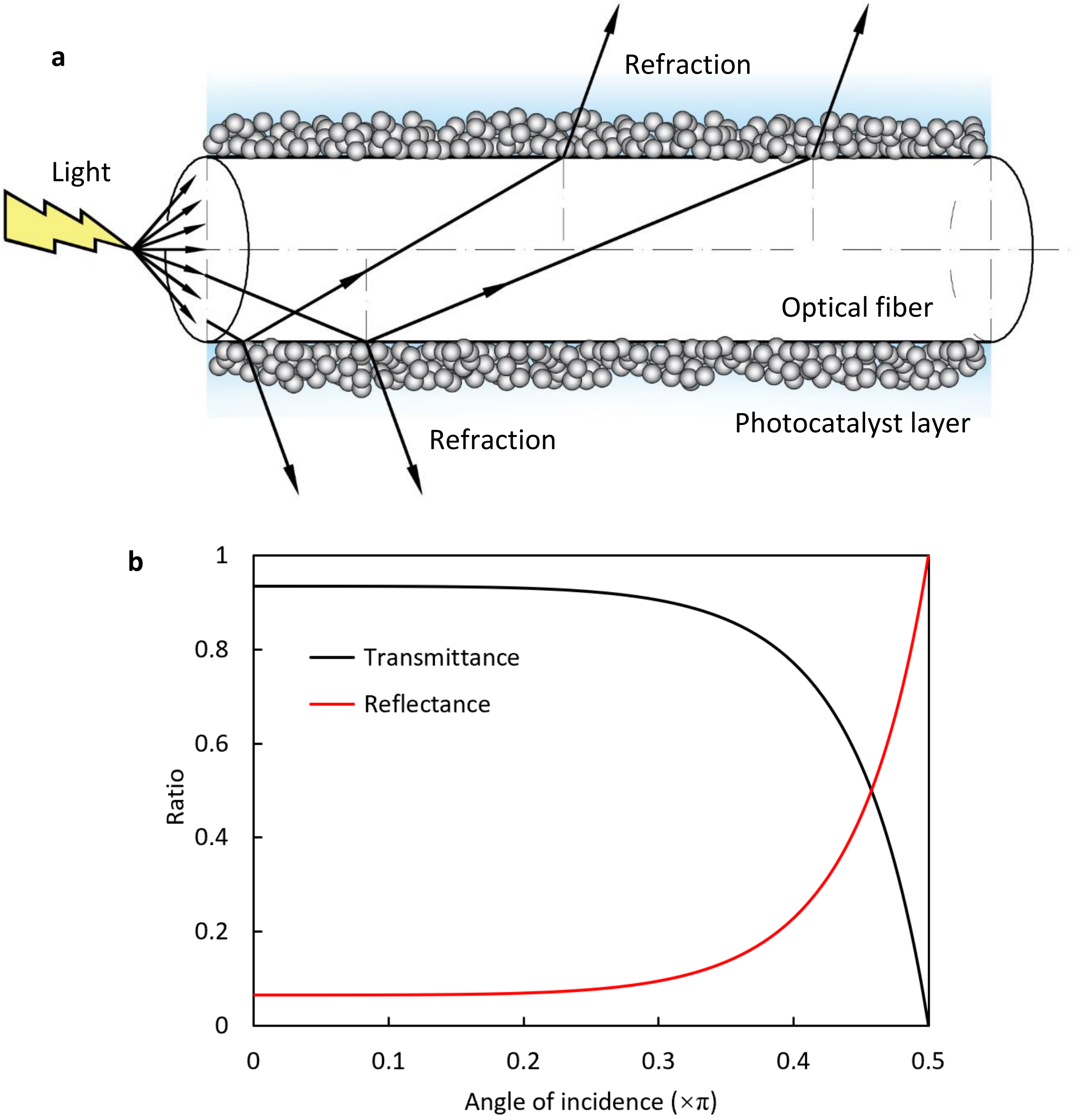

Supplementary Figure 1. Light propagation in photocatalyst-coated optical fibers coated with thick and dense photocatalyst layers. (a) Light delivering to P-OFs refracts out of the fiber and activate photocatalysts; (b) Fresnel transmittance/reflectance of the light refracted from optical fibers to photocatalyst layers as a function of the incident angle using TiO_2-coated quartz optical fibers as an example.

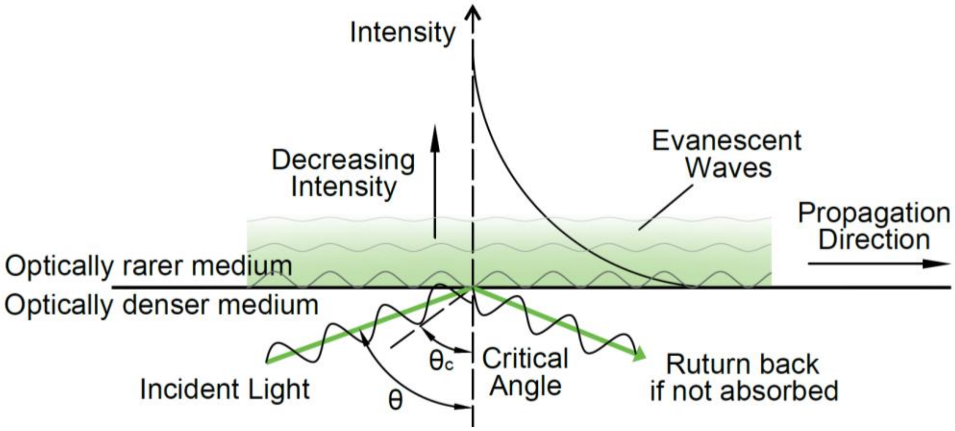

Supplementary Figure 2. Schematic of evanescent wave generation.

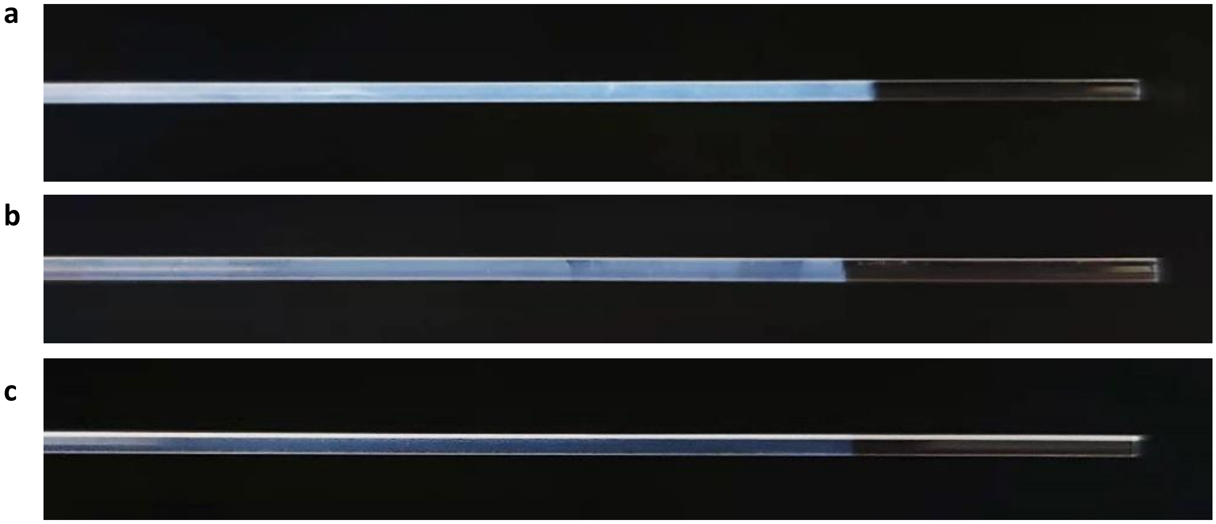

Supplementary Figure 3. Photos of TiO_2 layers of the three TiO_2-QOFs. (a) TiO_2-QOF-High coated with 81.8 μg/cm^2 TiO_2; (b) TiO_2-QOF-Med coated with 16.9 μg/cm^2 TiO_2; (c) TiO_2-QOF-Low coated with 4.8 μg/cm^2 TiO_2.

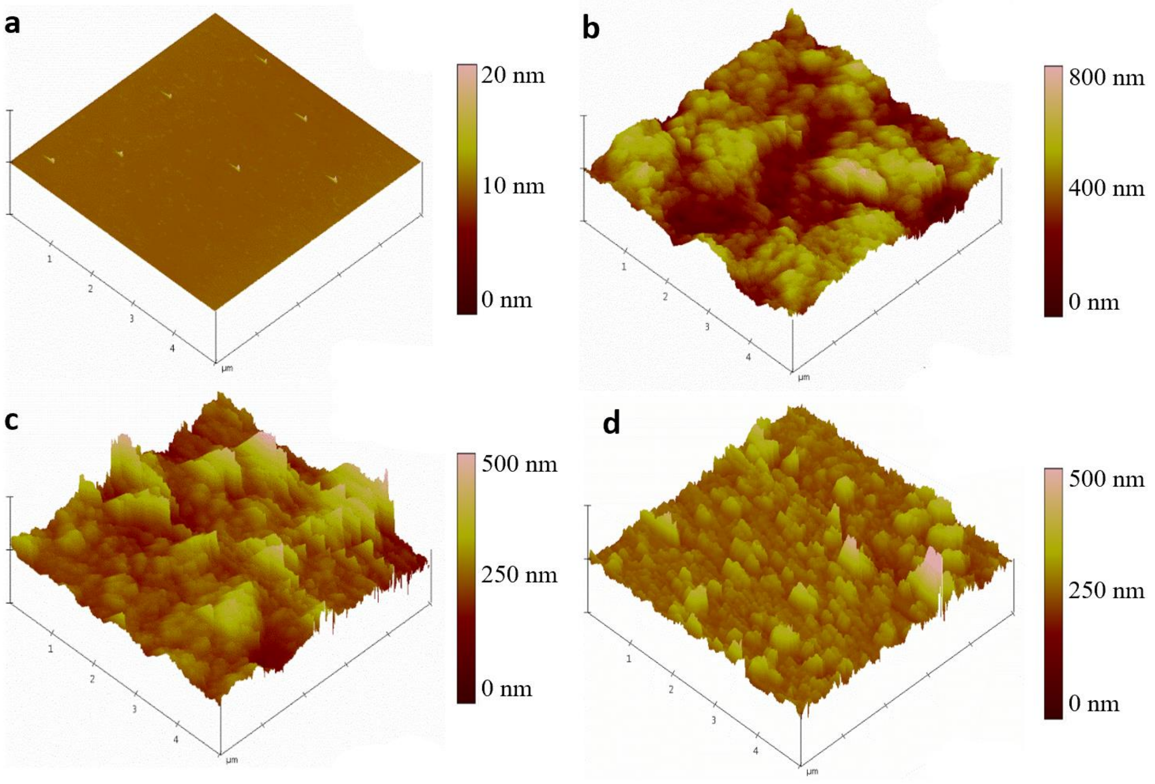

Supplementary Figure 4. Atomic Force Microscopy (AFM) images of the surfaces of uncoated optical fibers and the three TiO_2-QOFs. (a) Uncoated optical fibers; (b) TiO_2-QOF-High coated with 81.8 μg/cm^2 TiO_2; (c) TiO_2-QOF-Med coated with 16.9 μg/cm^2 TiO_2; (d) TiO_2-QOF-Low coated with 4.8 μg/cm^2 TiO_2.

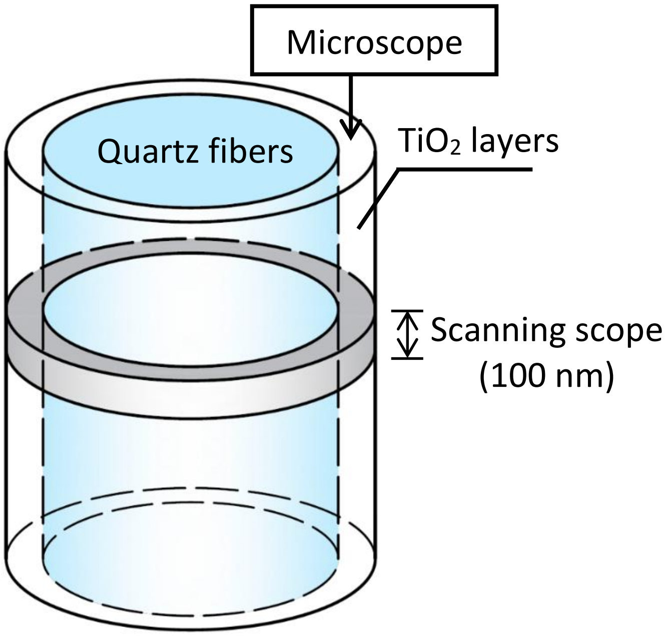

Supplementary Figure 5. Schematic of 3D optical profiler scanning methods.

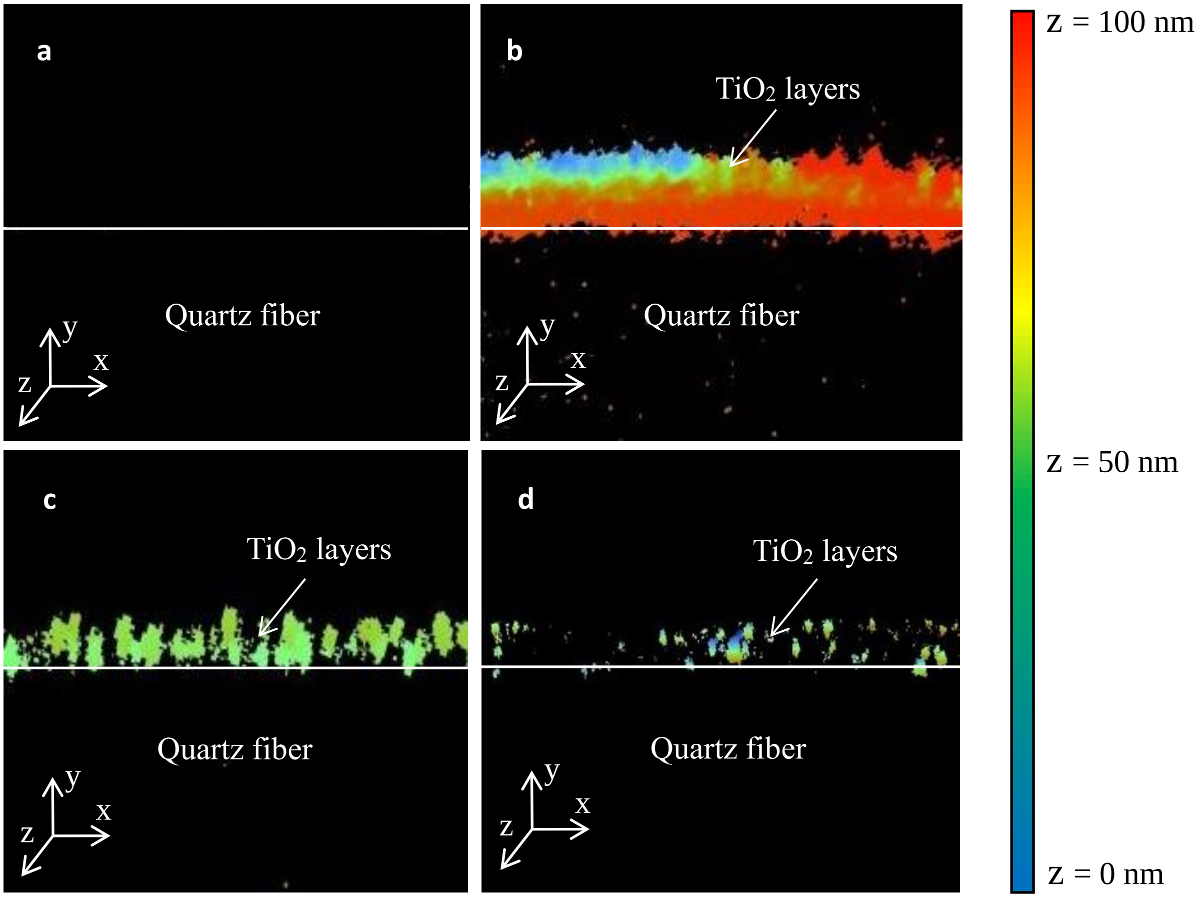

Supplementary Figure 6. 3D optical profiler images of the cross sectional views of (a) uncoated optical fibers; (b) TiO_2-QOF-High coated with 81.8 μg/cm^2 TiO_2; (c) TiO_2-QOF-Med coated with 16.9 μg/cm^2 TiO_2; and (d) TiO_2-QOF-Low coated with 4.8 μg/cm^2 TiO_2.

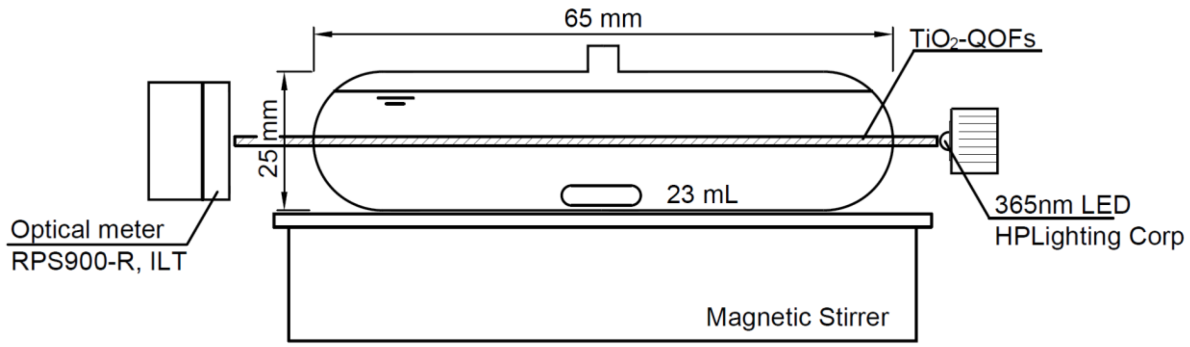

Supplementary Figure 7. Experimental setup.

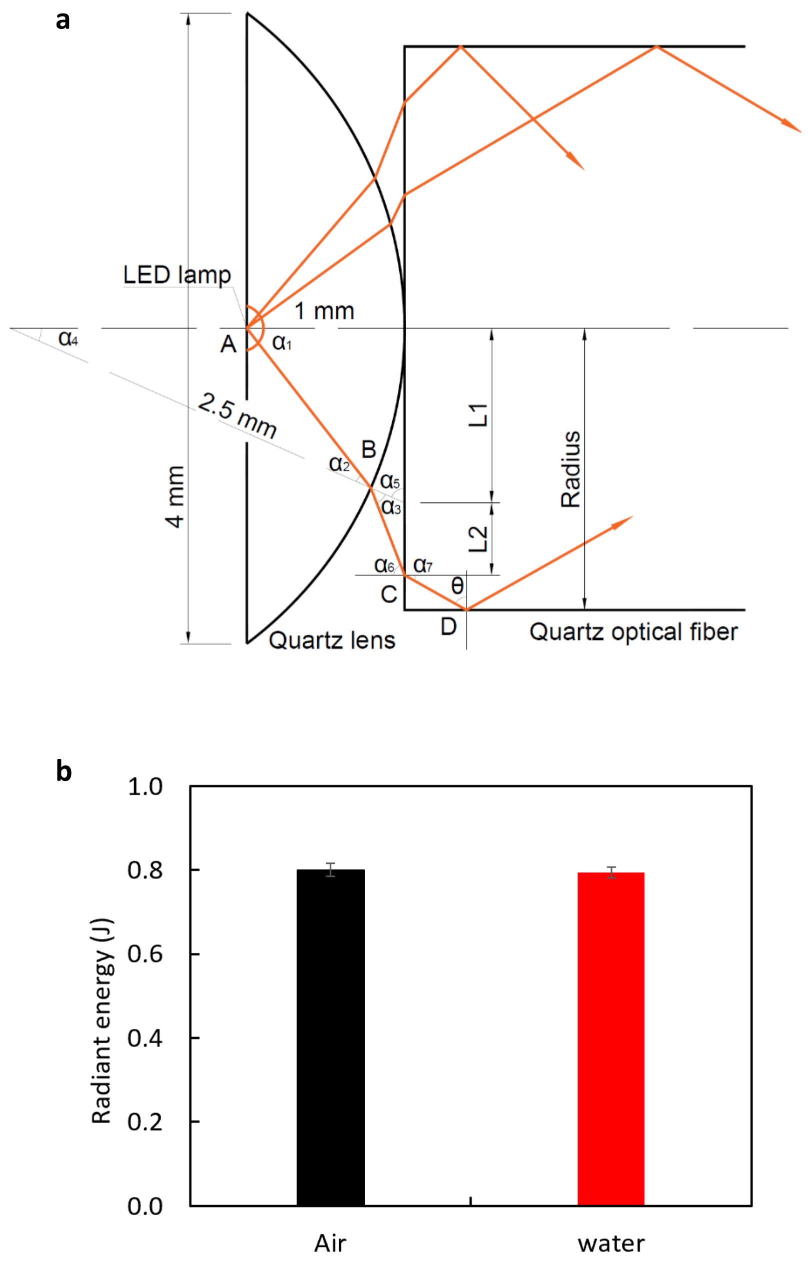

Supplementary Figure 8. (a) Schematic of light propagation from a LED lamp to a quartz optical fiber; (b) radiant energy measured at the terminal end of the uncoated quartz optical fibers.

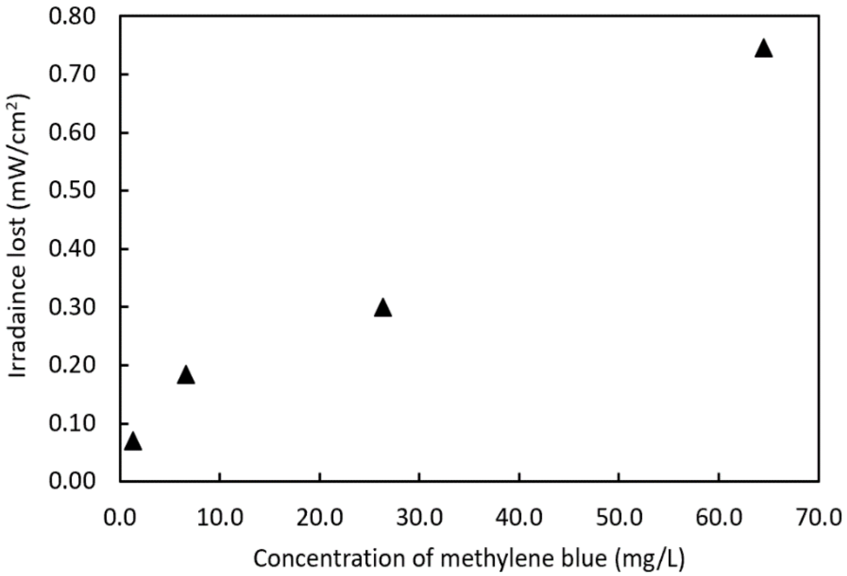

Supplementary Figure 9. Evanescent waves generated at uncoated optical fiber surface absorbed by methylene blue as a function of methylene blue concentrations. (Conditions: light intensity = 17.6 mW/cm^2, wavelength = 365 nm)

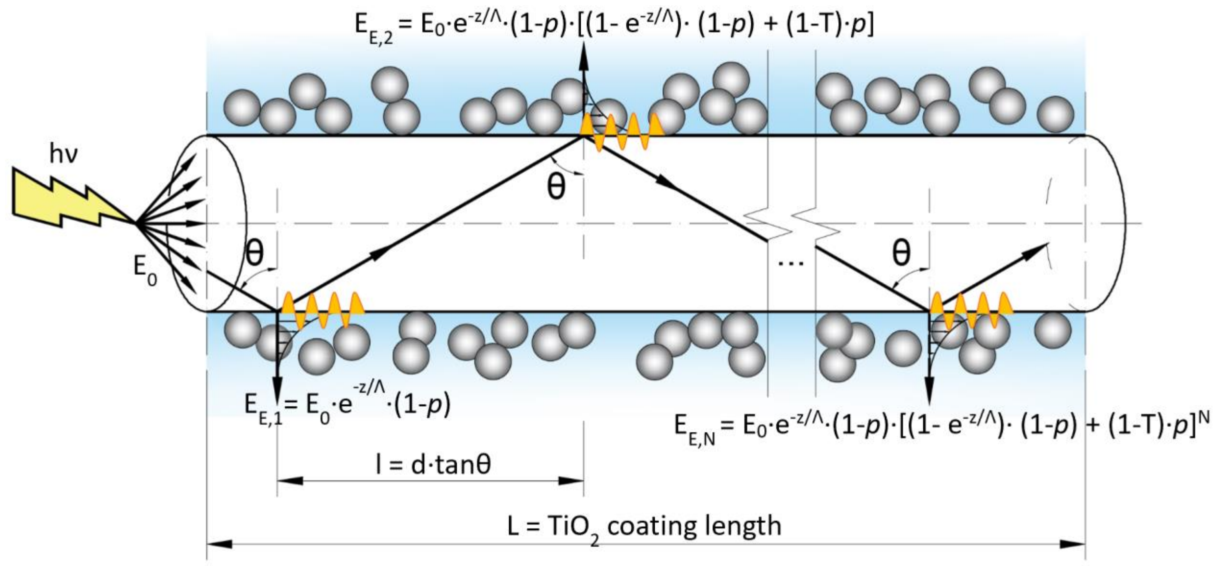

Supplementary Figure 10. Schematic of derivation of the radiant energy dissipation of evanescent waves in TiO_2-QOFs.

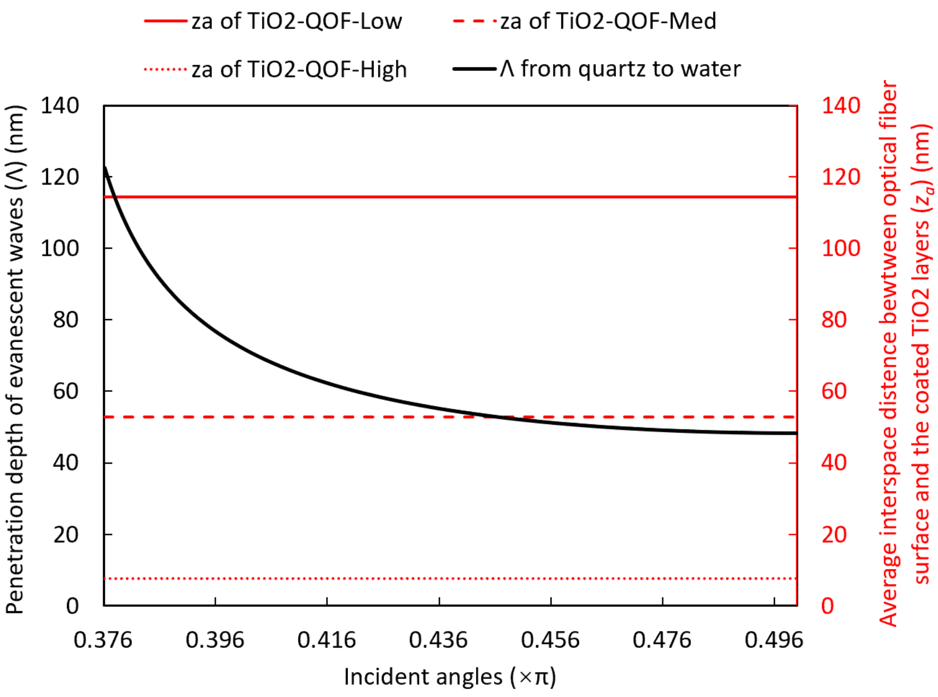

Supplementary Figure 11. Relationships between the penetration depth of evanescent waves (Λ) from quartz to water and the average interspace distance between the optical fiber surfaces and the coated TiO_2 layers (z_a) of the three TiO_2-QOFs. (Conditions: UV-LED intensity = 7 mW/cm^2, light wavelength = 365 nm)

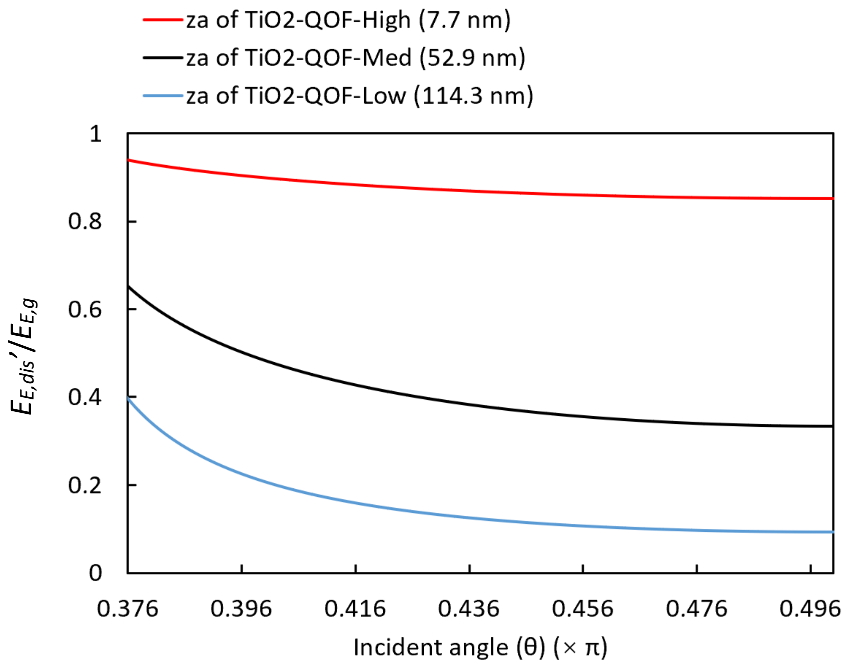

Supplementary Figure 12. The percentage of the radiant energy of evanescent waves dissipated into TiO_2 coating layers ($E_{E,dis}'$) to the generated radiant energy of evanescent waves ($E_{E,g}$) at each TIR spot as a function of light incident angles in the three TiO_2-QOFs at different z_a.

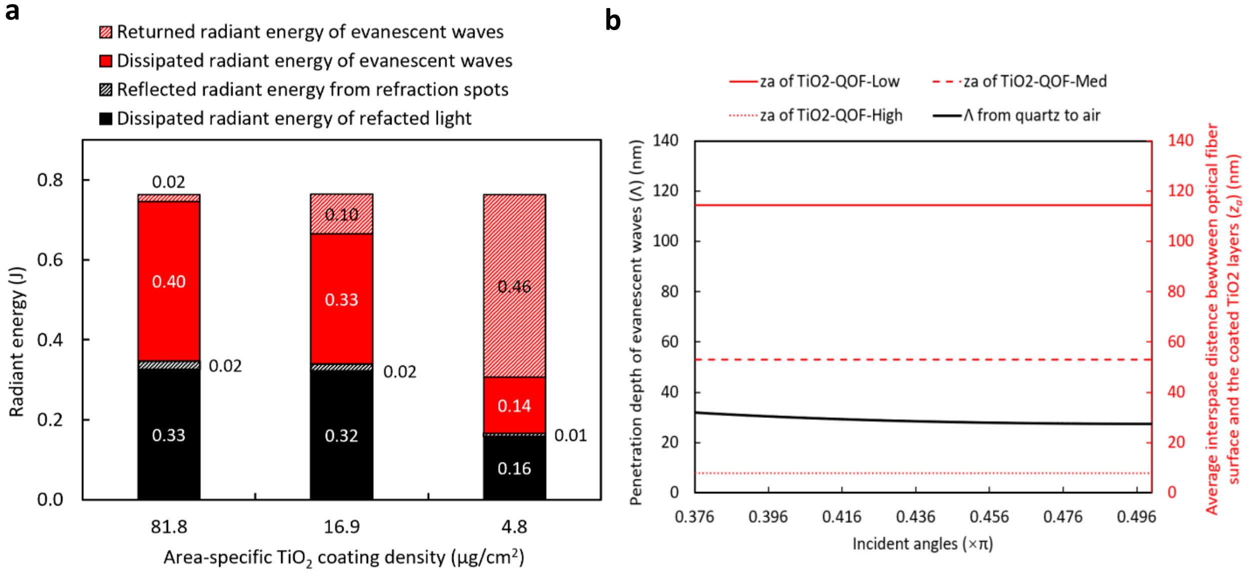

Supplementary Figure 13. Modeling results of light propagation in the three TiO_2-QOFs. (a) modeling results of the dissipated radiant energy of evanescent waves ($E_{E,dis}'$) and refracted light ($E_{R,dis}'$) as well as the returned radiant energy of evanescent waves ($E_{E,return}$) and the reflected radiant energy from refraction spots ($E_{R,reflect}$) when TiO_2-QOFs were exposed to air; (b) relationships between the penetration depth of evanescent waves (Λ) from quartz to air and the average interspace distance between the optical fiber surfaces and coated TiO_2 layers (z_a) of the three TiO_2-QOFs. (Conditions: light intensity = 7 mW/cm^2, wavelength = 365 nm, TiO_2 coating length = 6.5 cm, irradiation duration = 4 h)

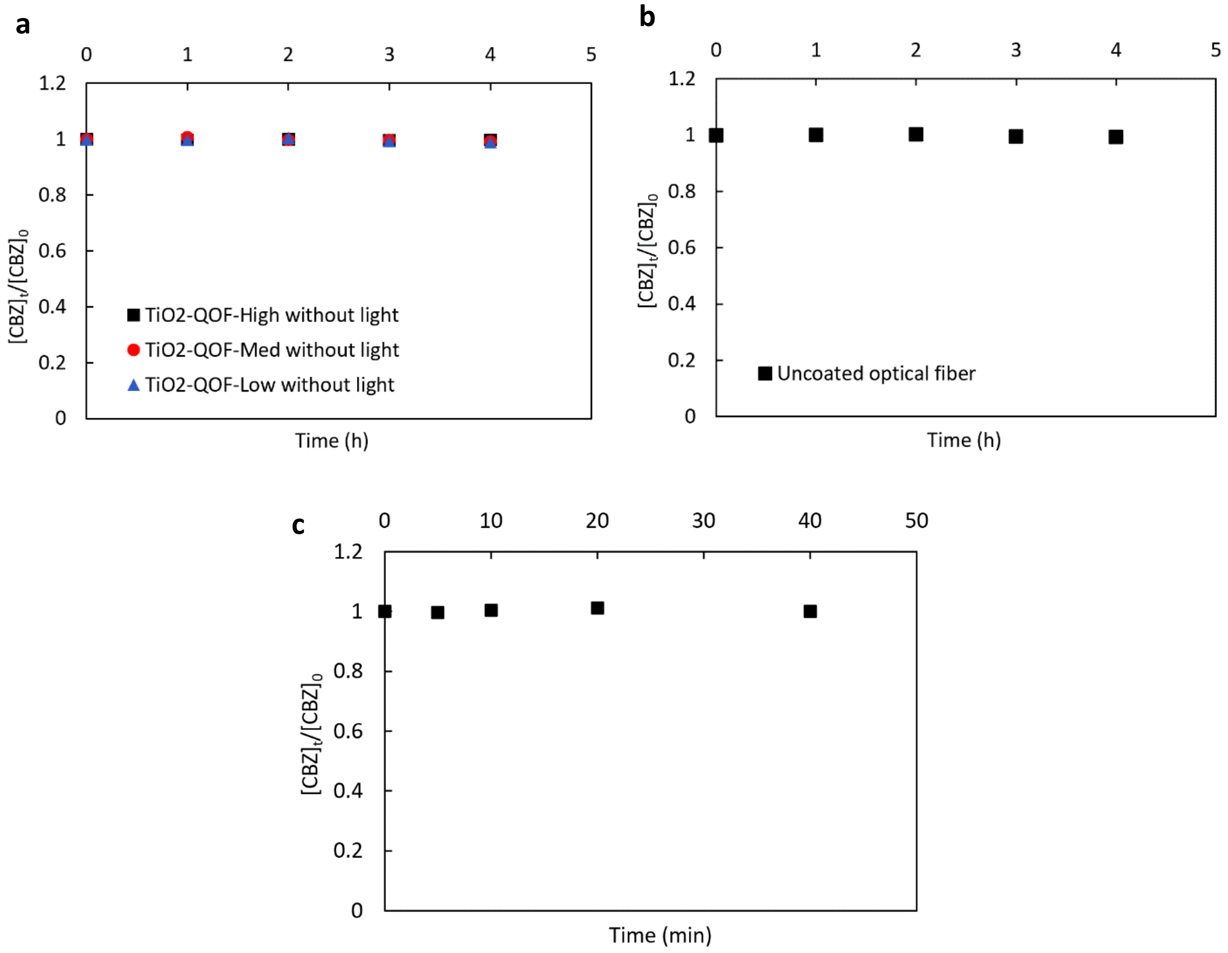

Supplementary Figure 14. Carbamazepine degradation by (a) the three TiO_2-QOFs without light irradiance, and (b) the uncoated optical fibers without TiO_2 coated under 365 nm UV (Conditions: UV-LED intensity = 7.02 mW/cm^2, light wavelength = 365 nm, $[CBZ]_0$ = 2 μM, volume =23 mL, reaction time = 4 h); and (c) carbamazepine adsorbed by TiO_2 suspension (Conditions: reactor volume = 100 mL, [CBZ] = 2 μM, TiO_2 concentration = 5 g/L).

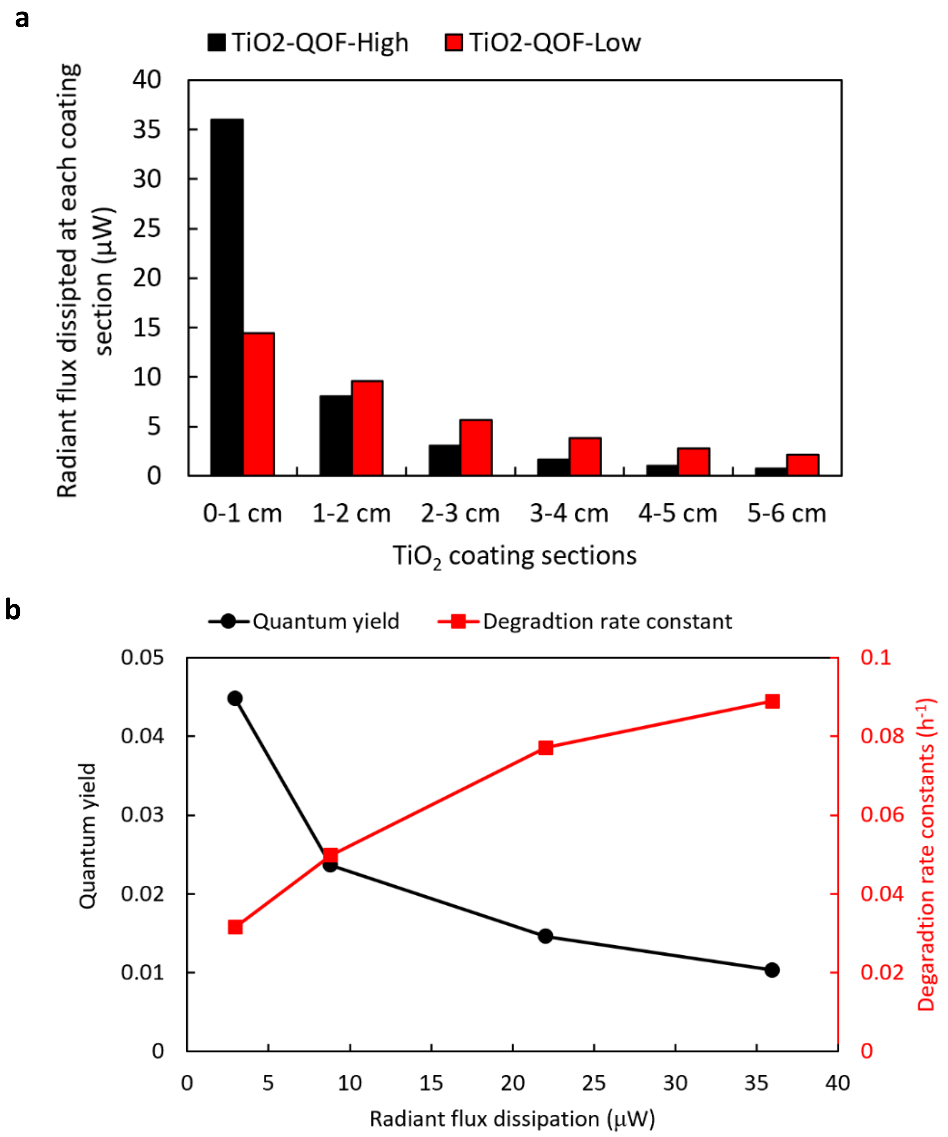

Supplementary Figure 15. (a) Radiant flux dissipated at each coating section at a length of 1 cm along TiO_2-QOF-High and TiO_2-QOF-Low at an incident light intensity of 7.02 mW/cm^2; (b) carbamazepine degradation by TiO_2-QOF-High at a coating length of 1mm as a function of radiant flux dissipation. (Conditions: light wavelength = 365 nm, initial carbamazepine concentration = 2 μM, irradiation duration = 4 h, reactor volume = 23 mL)

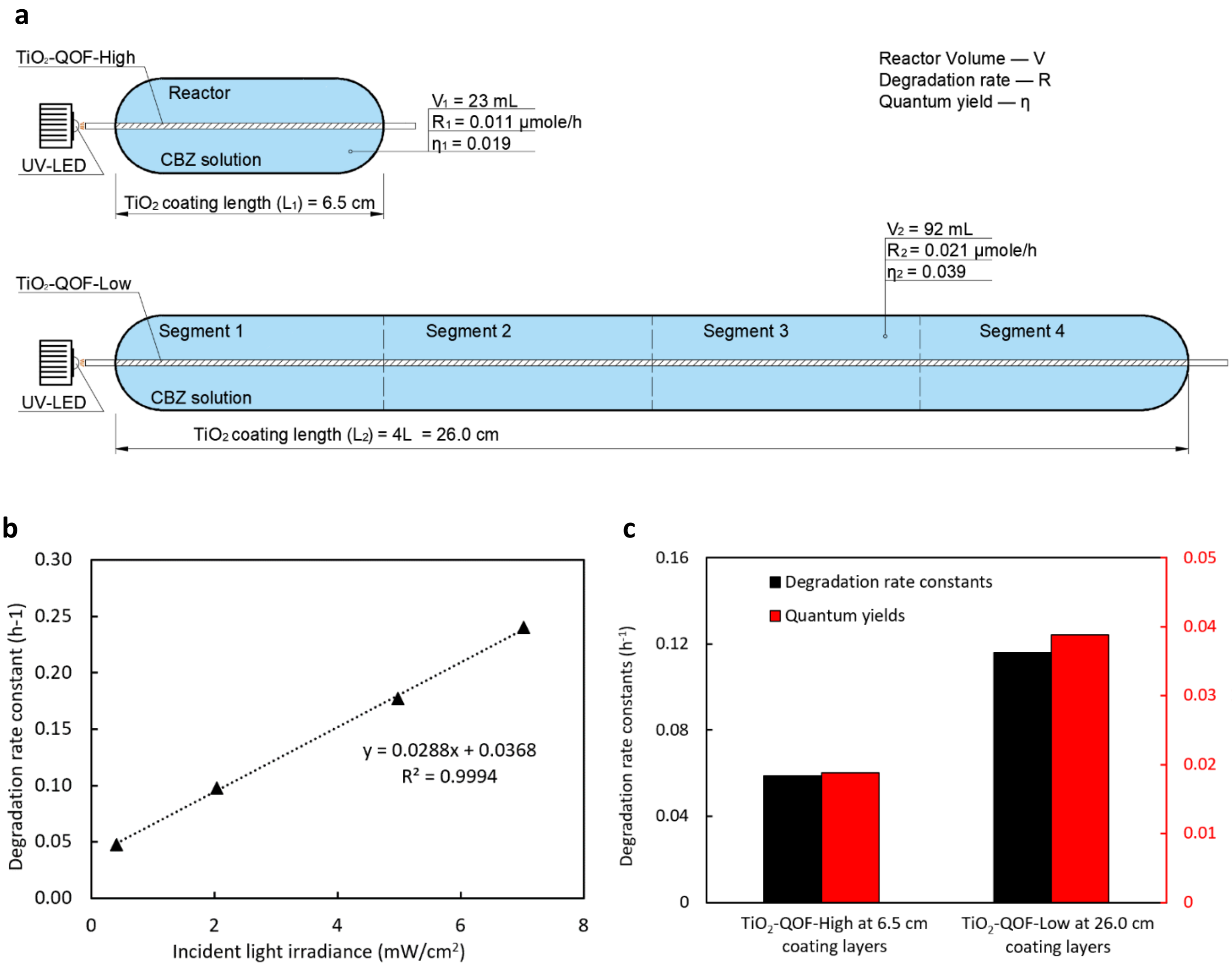

Supplementary Figure 16. (a) The reactors containing the TiO_2-QOF-High with 6.5 cm TiO_2 coating length and the TiO_2-QOF-Low at 26 cm coating length, respectively; (b) carbamazepine degradation by the TiO_2-QOF-Low at 6.5 cm coating length as a function of incident light irradiances (Conditions: light wavelength = 365 nm, $[CBZ]_0$ = 2 μM, volume =23 mL); (c) the comparison in carbamazepine degradation between the reactor containing the TiO_2-QOF-High at 6.5 cm coating length and the reactor containing the TiO_2-QOF-Low at 26 cm coating length in a 92 mL reactor.

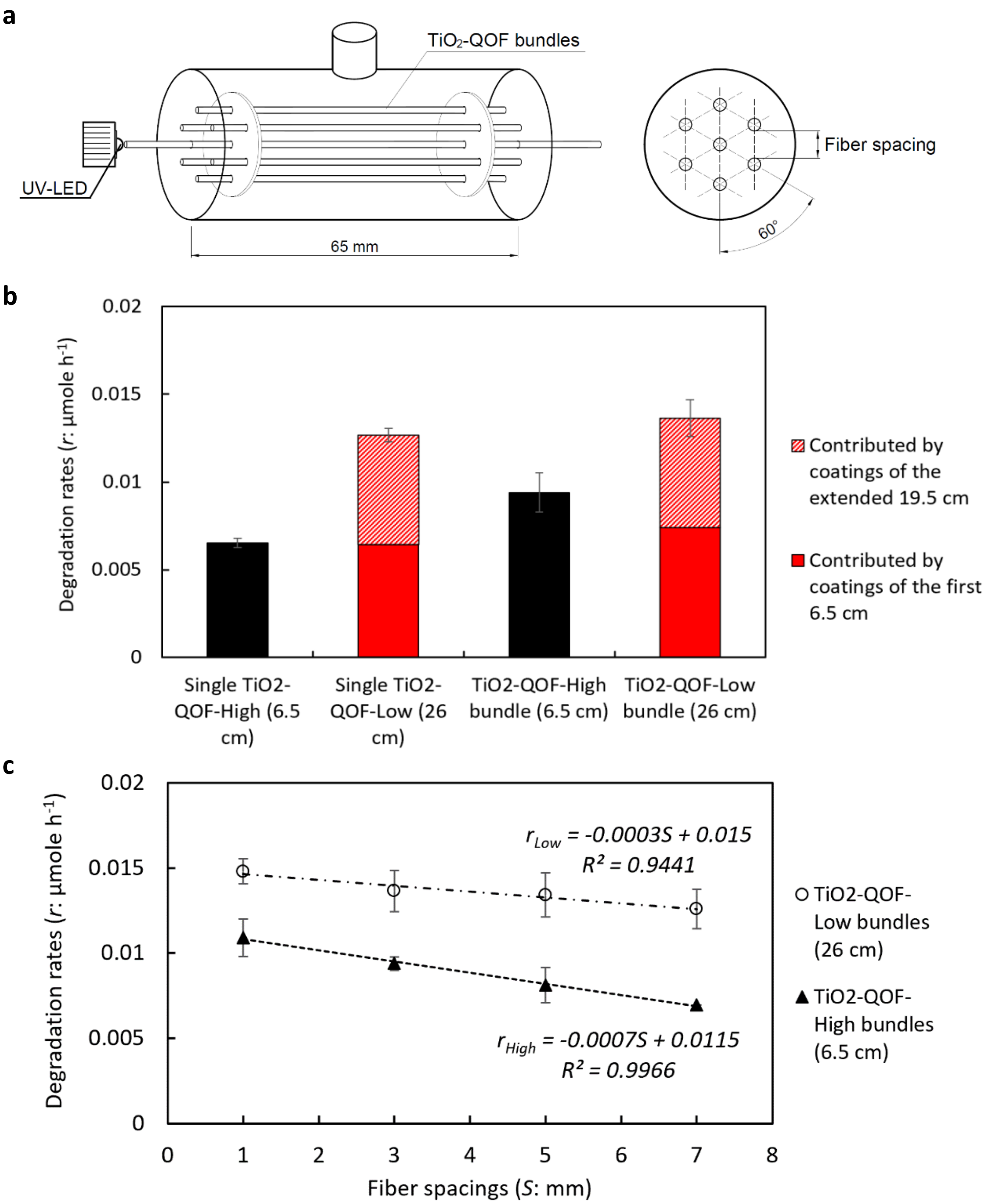

Supplementary Figure 17. (a) Schematics of a hexagonal arrangement for a TiO_2-QOF bundle which consists of one TiO_2-QOF in the center and six TiO_2-QOFs at the edge for carbamazepine degradation; (b) the comparison in carbamazepine degradation rates by a single fiber of TiO_2-

QOF-High (6.5 cm), a single fiber of TiO_2-QOF-Low (26 cm), a bundle of TiO_2-QOF-High (6.5 cm), and a bundle of TiO_2-QOF-Low (26 cm) irradiated by a UV-LED; (c) the carbamazepine degradation rates by a TiO_2-QOF-Low bundle irradiated by one UV-LED (r_{Low}) and that by a TiO_2-QOF-High bundle irradiated by one UV-LED (r_{High}) as a function of fiber spacings (S). (Conditions: light intensity = 7.02 mW/cm^2, wavelength = 365 nm, initial carbamazepine concentration = 2 μM, irradiation duration = 4 h).

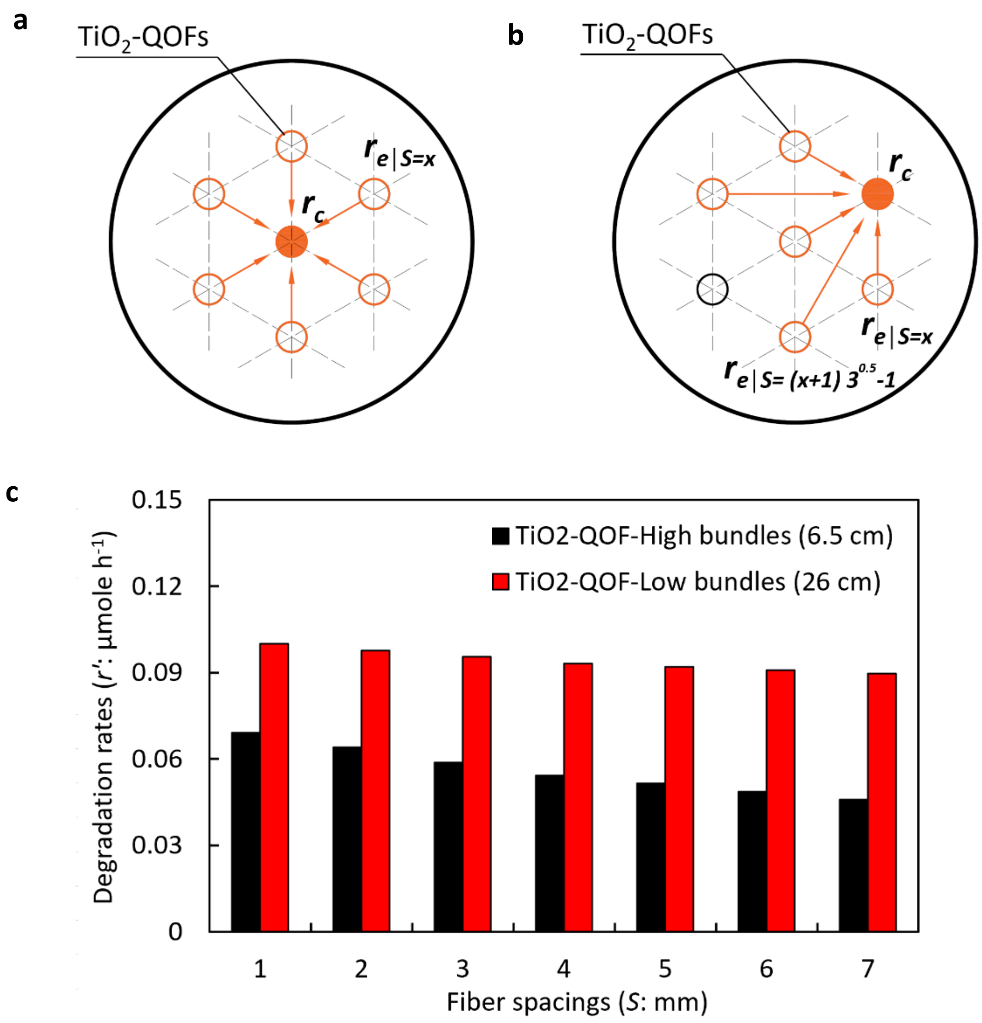

Supplementary Figure 18. Schematics of the crosse-section of a TiO_2-QOF bundle to simulate (a) the carbamazepine degradation rate by the TiO_2-QOF in the center, which equals to the sum of the carbamazepine degradation rate by this TiO_2-QOF irradiated by a UV-LED (r_c) and that by this TiO_2-QOF irradiated by refracted light from the surrounding six TiO_2-QOFs at a fiber spacing (S) of x ($6\times r_{e|S=x}$), and (b) the carbamazepine degradation rate by the TiO_2-QOF at the edge, which equals to the sum of r_c, $3\times r_{e|S=x}$, and $2\times r_{e|S=(x+1)3\text{^}0.5\text{-}1}$,; (c) the carbamazepine degradation rates by the TiO_2-QOF-Low bundle each irradiated by one UV-LED (r'_{Low}) and that by the TiO_2-QOF-High bundle each irradiated by one UV-LED (r'_{High}) as a function of S. (Conditions: light intensity of a UV-LED = 7.02 mW/cm^2, wavelength = 365 nm, initial carbamazepine concentration = 2 µM, irradiation duration = 4 h).

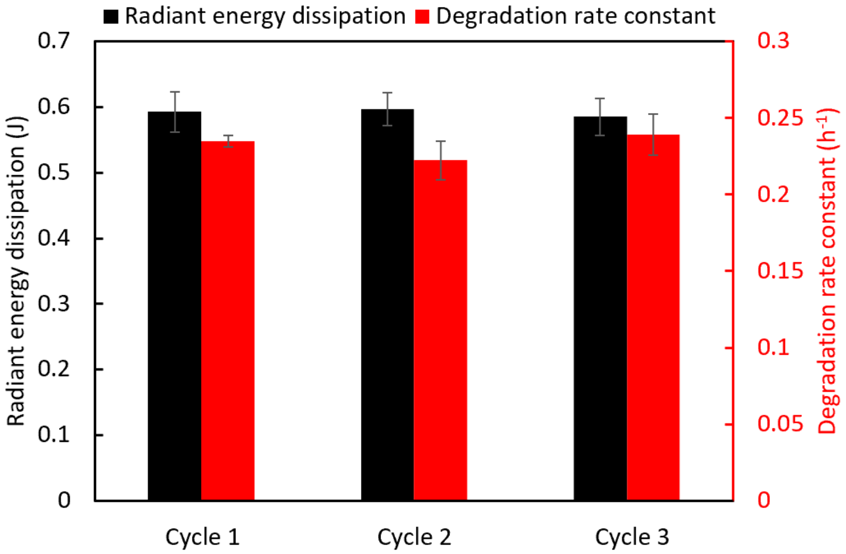

Supplementary Figure 19. Radiant energy dissipation and carbamazepine degradation rate constants of TiO_2-QOF-Low for each cycle. (Conditions: light intensity = 7.02 mW/cm^2, wavelength = 365 nm, TiO_2 coating length = 6.5 cm, irradiation duration = 4 h)

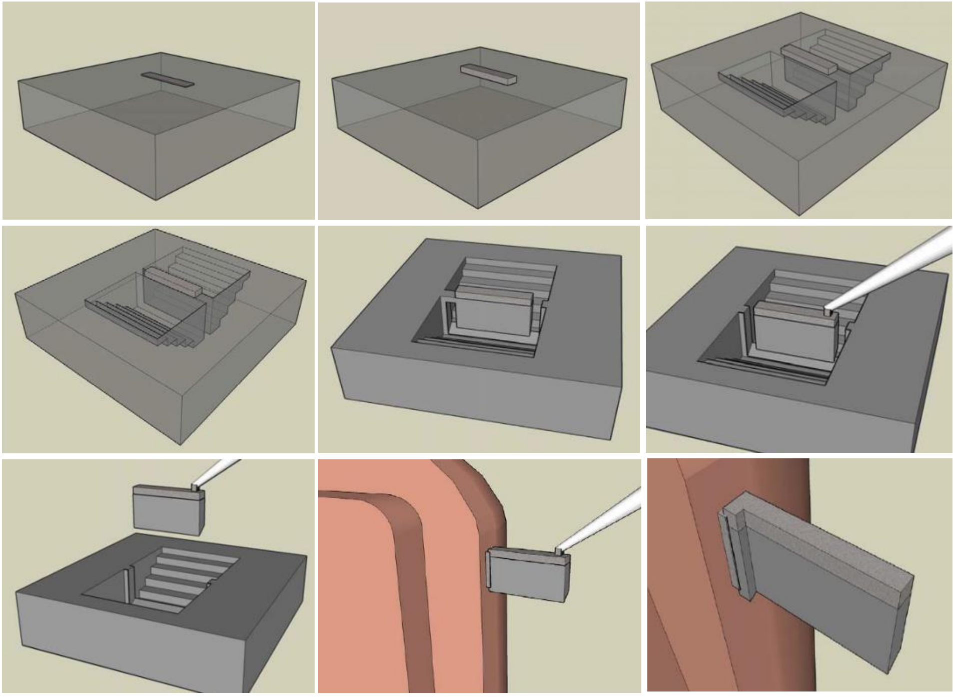

Supplementary Figure 20. Preparation procedures of TEM lamella for cross sections of TiO_2-QOFs using a FIB. (Images from Materials Characterization and Preparation Facility of the Hong Kong University of Science and Technology)

Supplementary Table 1. Experimental conditions for derivation of p and z_a values.

	Light intensity (mW/cm^2)	External medium of TiO_2-QOFs	TiO_2 coating length (cm)
Condition (1)	7.02	Air/water	6.5
Condition (2)	7.02	Air/ ethanol	6.5
Condition (3)	7.02	Air/water	5.0

Supplementary Table 2. Calculation results of p and z_a values

	Name	Condition (1)	Condition (2)	Condition (3)	Average	Stdev	Stdev/ Average
p	TiO_2-QOF-High	0.531	0.520	0.532	0.528	0.005	0.99%
	TiO_2-QOF-Med	0.198	0.209	0.210	0.206	0.005	2.64%
	TiO_2-QOF-Low	0.034	0.035	0.032	0.034	0.001	3.63%
z_a	TiO_2-QOF-High	7.900	7.748	7.528	7.725	0.153	1.97%
	TiO_2-QOF-Med	52.575	53.192	52.856	52.874	0.252	0.48%
	TiO_2-QOF-Low	115.460	118.342	109.083	114.295	3.868	3.38%

Supplementary Table 3. The comparison of calculated radiant energy dissipation and measured radiant energy dissipation in the conditions of different incident light irradiance.

Name	p & z_a value	Irradiance (mW/cm^2)	Measured $E_{dis,air}$ (J)	Calculated $E_{dis,air}$ (J)	**Error**	Measured $E_{dis,water}$ (J)	Calculated $E_{dis,water}$ (J)	**Error**
TiO_2-QOF-High	p = 0.528; z_a = 7.7 nm	7.02	0.737	0.725	**-1.71%**	0.750	0.734	**-2.11%**
		4.98	0.508	0.517	**1.79%**	0.530	0.521	**-1.67%**
		2.04	0.212	0.212	**0.18%**	0.218	0.214	**-1.80%**
		0.41	0.042	0.042	**1.89%**	0.043	0.043	**-1.09%**
TiO_2-QOF-Med	p = 0.206; z_a = 52.9 nm	7.02	0.629	0.648	**2.96%**	0.707	0.686	**-3.10%**
		4.98	0.445	0.456	**2.56%**	0.505	0.498	**-1.57%**
		2.04	0.184	0.187	**1.88%**	0.206	0.204	**-0.81%**
		0.41	0.036	0.037	**2.78%**	0.041	0.041	**-0.20%**
TiO_2-QOF-Low	p = 0.034; z_a = 114.3 nm	7.02	0.295	0.299	**1.28%**	0.583	0.582	**-0.10%**
		4.98	0.203	0.212	**3.97%**	0.406	0.402	**-0.87%**
		2.04	0.087	0.087	**-0.31%**	0.164	0.165	**0.97%**
		0.41	0.017	0.017	**1.04%**	0.034	0.033	**-2.43%**

Remarks: $Error = \frac{Calculated\ E_{dis} - Measured\ E_{dis}}{Calculated\ E_{dis}} \times 100\%$

Supplementary Table 4. Porosity (ε), thickness (δ), tortuosity (τ), Thiele modulus (ϕ) and the internal effectiveness factor (η) of the three TiO_2-QOFs

Name	Porosity (ε)	Thickness (δ: nm)	Tortuosity (τ)	Thiele modulus (ϕ)	Internal effectiveness factor (η)
TiO_2-QOF-High	0.622	507.51	1.208	1.20×10^{-4}	1.00
TiO_2-QOF-Med	0.71	136.31	1.157	2.89×10^{-5}	1.00
TiO_2-QOF-Low	0.896	104.72	1.072	1.95×10^{-5}	1.00

Supplementary Table 5. Dip-coating conditions and TiO_2 coating parameters of the five TiO_2-QOFs (Conditions: light intensity = 7.02 mW/cm^2, wavelength = 365 nm, TiO_2 coating length = 6.5 cm, irradiation duration = 4 h).

Name	Dip-coating conc. (mg/L)	Dipping duration (min)	Coating cycles	*p*	z_a (nm)	Degradation rate constants (h^{-1})	Quantum yields
TiO_2-QOF-High	10,000	0.5	5	0.528	7.725	0.237	0.0189
TiO_2-QOF-Med	10,000	0.5	1	0.206	52.874	0.229	0.0195
TiO_2-QOF-Low	40	60	1	0.034	114.295	0.240	0.0248
TiO_2-QOF-Low’	40	30	1	0.026	127.973	0.120	0.0138
TiO_2-QOF-Low’’	20	30	1	0.018	139.500	0.076	0.0093

Supplementary Table 6. Quantum yields of carbamazepine degradation by UV/Chlorine and UV/H_2O_2 process

Process	Quantum yield	Wavelength	Irradiance	$[CBZ]_0$	Reference
UV/chlorine	0.0232	254 nm	0.0286×10^{-6} Einstein/s	3.98 μM	[8]
	0.00056	254 nm	0.3318×10^{-6} Einstein/s	2.1 μM	[9]
UV/H_2O_2	0.0087	254 nm	0.0286×10^{-6} Einstein/s	3.98 μM	[8]
	0.00441	254 nm	671.2×10^{-6} Einstein	4.23 μM	[10]

Supplementary Notes

Note 1. Light propagation in photocatalyst-coated optical fibers

Supplementary Figure 1(a) shows the light propagation in the photocatalyst-coated optical fibers (P-OFs) coated with thick and dense photocatalyst layers. When light propagates down the P-OFs, light in optical fibers is refracted into the coated photocatalyst layers and activates photocatalysts, because the refractive index of photocatalyst layers is higher than that optical fiber. Supplementary Figure 1(b) shows transmittance/reflectance of the light refracted from a fiber core to photocatalyst coating layers as a function of the incident angle using TiO_2-coated quartz optical fibers as an example calculated using Fresnel equations[1,2]. The transmittance is quantified as the fraction of the incident light refracted into TiO_2 coating layers from quartz fibers, while the reflectance is quantified as the fraction of the incident light reflected from the quartz/TiO_2 interface. The sum of transmittance and reflectance is equal to 1. As shown in Supplementary Figure 1(b), over 80% of the incident light at angles from 0 to 0.4π in quartz fibers are refracted out into TiO_2 coating layers, while less than 20% of which is reflected and continues to propagate along the quartz optical fiber. With increasing angles from 0.4π to 0.5π, the transmittance decreases significantly.

Note 2. Evanescent waves generation from total internal reflection (TIR)

Light is totally reflected when it propagates from an optically denser medium to an optically rarer medium, at an angle (θ) greater than critical angle (θ_c) as derived from Snell's law[1,2]:

$$\theta_c = sin^{-1}(n_r/n_d) \quad (1)$$

where n_r is the refractive index of the optically rarer medium, and n_d is the refractive index of the optically denser medium. Meanwhile, a small portion of the reflected light penetrates through the interface and forms evanescent waves, which propagate parallel to the interface with a very short distance called penetration depth of evanescent waves (Λ), as shown in Eq. 2[3–5].

$$\Lambda = \frac{\lambda}{4\pi} \cdot ({n_q}^2 \cdot sin^2\theta - {n_e}^2)^{-1/2} \quad (2)$$

where λ is the wavelength of the radiation source. The intensity of evanescent waves (I) decays exponentially with vertical distances perpendicular to the interface (z) as shown in Eq. 3:

$$I = I_0 \cdot e^{-\frac{z}{\Lambda}} \quad (3)$$

where I_0 is the irradiance at the interface. The evanescent wave energy, if not absorbed by the external medium, will eventually return to the original medium[2,6].

Note 3. Characterization of TiO_2-QOFs.

A. Photos of TiO_2 layers on quartz optical fibers

The photos of the three TiO_2-QOFs are shown in Supplementary Figure 3. TiO_2-QOF-High was fabricated by dipping optical fibers in TiO_2 suspension at 10,000 mg/L for 0.5 min and air drying for another 0.5 min with five consecutive dip-coating/drying cycles. Due to high TiO_2 dip-coating concentration and five dip-coating/drying cycles, TiO_2-QOF-High is coated with 81.8 $\mu g/cm^2$ TiO_2 and shows a white and opaque TiO_2 coating layer. Reducing the dip-coating/drying cycle from five to one for TiO_2-QOF-Med, the area-specific TiO_2 coating density decreases by 79.3% From TiO_2-QOF-High to TiO_2-QOF-Med and the TiO_2 layers becomes less opaque. By decreasing the TiO_2 dip-coating concentration from 10,000 mg/L to 40 mg/L and dip-coating for a longer time (1 h) with one dip-coating/drying cycle for TiO_2-QOF-Low, only 4.8 $\mu g/cm^2$ TiO_2 is coated on the surfaces of optical fibers and the TiO_2 layer in TiO_2-QOF-Low is almost transparent.

B. Atomic Force Microscopy (AFM) images of the surfaces of TiO_2-QOFs

Supplementary Figure 4 shows the AFM images of the surfaces of uncoated optical fibers without any coating and the three TiO_2-QOFs. The height of the TiO_2 coating layers in each figure is indicated in the color scale. The image of the surfaces of uncoated optical fibers (Supplementary Figure 4(a)) is like a flat sheet, and has no color difference, proving that there is no TiO_2 coated on the surface. In contrast, Supplementary Figure 4(b) shows the surface of TiO_2-QOF-High where there is a big height difference, suggesting TiO_2 nanoparticles cluster together and form a thick and dense TiO_2 coating layer. With decreasing TiO_2 coated on optical fibers, fewer TiO_2 nanoparticles cluster together in TiO_2-QOF-Med and thus some more flat and uniform coating areas appear. In TiO_2-QOF-Low, with less amount of TiO_2 coated, TiO_2 nanoparticles are evenly spread on the surface of the optical fiber to form a thin and porous structures.

C. 3D optical profiler images of the cross sectional views of TiO_2-QOFs

The cross sectional views of the uncoated optical fibers and three TiO_2-QOFs are characterized by a 3D optical profiler as shown in Supplementary Figure 5. The TiO_2-QOFs were placed upright and cross sections were scanned using the white light interferometry from top to bottom at a scope of 100 nm to obtain the images of TiO_2 layers. The images of the cross sectional views of the uncoated optical fibers and the three TiO_2-QOFs are shown in Supplementary Figure 6. Since the scanning is from up to bottom, the obtained images with red color represent a higher location (z) in the scope of 100 nm of scanning length, while those with blue color represent a lower location. It is shown that there is no TiO_2 coated on the surface of uncoated optical fibers (Supplementary Figure 6(a)). However, Supplementary Figure 6(b) shows that there is a thick and dense TiO_2 coating layer on the surface. With decreasing TiO_2 coated on optical fibers, TiO_2-QOF-Med coated

with 16.9 μg/cm^2 TiO_2 shows a discrete structure of TiO_2 particles and has some surfaces areas without TiO_2 attached as shown in Supplementary Figure 6(c). In TiO_2-QOF-Low as shown in Supplementary Figure 6(d), the TiO_2 attachment is further reduced and the TiO_2 coating layer is the most discrete one compared with TiO_2-QOF-High and TiO_2-QOF-Med.

Note 4. Experimental setup

The photocatalytic experiments were conducted in a cylindrical glass reactor (23 mL) with a length of 65 mm and a diameter of 25mm. A single TiO_2-QOF was fixed in the reactor with one end mounted to a 365 nm LED (H44LV1C0, HPLighting) and the other connected to an optical meter (RPS900-R, International Light Technologies) as shown in Supplementary Figure 7. A magnetic stirrer (F203A0160, VELP) was placed at the bottom for solution stirring. All experiments were conducted at room temperature (25°C).

Note 5. Modeling of light propagation in TiO_2-QOFs.

A. Determination of the light incident angles

Supplementary Figure 8(a) shows the light rays emitted from an LED lamp are diffused by a lens and then transmitted into the quartz optical fiber directly connected to the lens. In order to determine the incident angles of light at the fiber interface (θ), Snell's law and geometric optics were applied. As shown in Supplementary Figure 8(a), the LED lamp is considered as a point light source (point A) with a quartz lens covered. The height of the lens is 1.0 mm and the diameter of the lens is 4.0 mm. Assuming the arc of the lens is a part of a circle, the distance from the arc to the center of the circle is 2.5 mm. The light rays are emitted to the surroundings and the angle between the light ray and the normal is defined as the diffusion angle (α_1). Taking one light ray as

an example, the light ray could further approach the surface of the lens (Point B), the ray could be refracted into air. The angles of incidence (α_2) is derived from law of sines (Eq. 4).

$$\alpha_2 = \sin^{-1}\left(\frac{1.5}{2.5} \cdot sin\, \alpha_1\right) \quad (4)$$

The angles of refraction (α_3) is derived from Snell's law (Eq. 5).

$$\alpha_3 = \sin^{-1}\left(\frac{1.5 \cdot n_q}{2.5} \cdot sin\, \alpha_1\right) \quad (5)$$

where n_q is the refractive index of the quartz. The light ray then could further reach the cross sections of quartz optical fibers (Point C) if assuming the radius of optical fibers (L) is big enough, the ray will be refracted into quartz optical fiber and then reach the Point D on side surfaces of the optical fibers. The angles of incidence at point C (α_6) is determined as shown in Eq. 6 using the geometric optics.

$$\alpha_6 = \frac{\pi}{2} - (\alpha_5 - \alpha_3) = \frac{\pi}{2} - \left(\left(\frac{\pi}{2} - \alpha_4\right) - \alpha_3\right) = \frac{\pi}{2} - \left(\left(\frac{\pi}{2} - (\alpha_1 - \alpha_2)\right) - \alpha_3\right)$$

$$= \alpha_1 - sin^{-1}\left(\frac{1.5}{2.5} \cdot sin\, \alpha_1\right) + sin^{-1}\left(\frac{1.5 \cdot n_q}{2.5} \cdot sin\, \alpha_1\right) \quad (6)$$

The angles of refraction at point C (α_7) is obtained according to Snell's law. Therefore, the relationship between α_1 and θ are obtained as shown in Eq. 7.

$$\theta = \frac{\pi}{2} - \alpha_7 = \frac{\pi}{2} - sin^{-1}\left\{\frac{sin\left[\alpha_1 - sin^{-1}\left(\frac{1.5}{2.5} \cdot sin\, \alpha_1\right) + sin^{-1}\left(\frac{1.5 \cdot n_q}{2.5} \cdot sin\, \alpha_1\right)\right]}{n_q}\right\} \quad (7)$$

On the other hand, to allow light rays to be delivered to optical fibers, the point C must be within the range of cross sections of quartz optical fibers, which means the sum of $L1$ and $L2$ should not be bigger than the radius of optical fibers (L). Therefore, the relationship between the radius of optical fibers (L) and α_1 could be established as shown in Eqs. 8-10.

$$L \geq L1 + L2 \qquad (8)$$

$$L1 = 2.5 \cdot tan\left[\alpha_1 - sin^{-1}\left(\frac{1.5}{2.5} \cdot sin\,\alpha_1\right)\right] \qquad (9)$$

$$L2 = \frac{1.5 \cdot n_q \cdot sin\,\alpha_1}{sin\left[\frac{\pi}{2} - \alpha_1 + sin^{-1}\left(\frac{1.5}{2.5} \cdot sin\,\alpha_1\right) - sin^{-1}\left(\frac{1.5 \cdot n_q}{2.5} \cdot sin\,\alpha_1\right)\right]} \cdot \left\{\frac{1}{sin\left[\frac{\pi}{2} - \alpha_1 + sin^{-1}\left(\frac{1.5}{2.5} \cdot sin\,\alpha_1\right)\right]} - 1\right\} \qquad (10)$$

The radius of optical fibers (L) is 0.5 mm, so α_1 is determined to be from 0 to 0.146π according to Eqs. 8-10. Therefore, θ is determined to be from 0.376π to 0.500π according to Eq. 7 if the quartz optical fiber is long enough. However, due to limited length of the TiO_2-QOFs (6.5 cm in this study), not all ray strikes the inner surfaces of the optical fiber, but a small portion of rays directly emitted out from the terminal end. θ which is directly emitted out from the terminal end is calculated using Eq. 11. The

$$\theta \geq \tan^{-1}\left(\frac{6.5}{L1 + L2}\right) \qquad (11)$$

The smallest θ which is directly emitted out from the terminal end without striking the inner surfaces of optical fiber is thus equal to 0.495π. Therefore, θ is determined to be from 0.376π to 0.495π if the TiO_2-QOFs have the length of 6.5 cm.

To verify the calculation result of θ, the radiant energy measured at the terminal end of uncoated optical fibers without any coating was measured. The critical angle (θ_c) of total internal reflection (TIR) at the quartz/air interface is 0.240π, while θ_c at the quartz/water interface is 0.365π. This means if θ of light covers the range from 0.240π to 0.365π, this portion of light that is totally reflected at the quartz/air interface is refracted out as the external medium changes from air to

water, and thus leads to radiant energy dissipation. However, if θ of light is always greater 0.365π, the radiant energy measured at the terminal end of uncoated optical fibers should be the same as the external medium changes from air to water. According to our calculation results, θ is from 0.376π to 0.500π which is greater 0.365π, so the radiant energy measured at the terminal end of uncoated optical fibers should be the same when the optical fiber is surrounded by air and water, respectively. Supplementary Figure 8(b) shows the radiant energy measured at the terminal end of uncoated optical fibers from a UV-LED at 7.02 mW/cm^2. It shows that the radiant energy measured at the terminal end of a uncoated optical fiber was the same when the uncoated optical fiber is surrounded by air and water, respectively, which supports our calculation result of θ.

B. Existence of evanescent waves on quartz optical fiber surfaces

The existence of evanescent waves on quartz optical fiber surfaces was demonstrated by tracking the irradiance loss in a UV irradiated uncoated fiber immersed in methylene blue (MB) solutions as shown in Supplementary Figure 9. When launching UV light to the uncoated optical fiber, the irradiance loss increased by around 10 times with increasing MB concentrations from 1.3 to 64.5 mg/L. The irradiance loss was due to the absorption of evanescent waves generated at uncoated quartz fiber surfaces by MB. This test proves the presence of evanescent waves.

C. Derivation of the radiant energy dissipation of evanescent waves and refracted light

A mathematical model was developed to simulate the radiant energy dissipation in different TiO_2-QOFs to quantify their light propagation in the forms of refracted light and evanescent waves. The model simulates the TIR and refraction of light at multiple spots along TiO_2-QOFs at a long distance irradiated by light with various angles. To obtain the radiant energy dissipation of

evanescent waves and refracted light in different TiO_2-QOFs, several assumptions are proposed before mathematical calculation:

a) Since the activation of TiO_2 involves both the refracted light and evanescent waves, the radiant energy dissipated in TiO_2-QOFs (E_{dis}) is the sum of radiant energy dissipation caused by refracted light ($E_{R,dis}'$) and evanescent waves ($E_{E,dis}'$);

b) TiO_2 layer structure on the optical fiber surface is stable and would not change during the time and during experiments such as changing the external medium of TiO_2-QOFs;

c) the light wave is considered to be geometric rays since the diameter of optical fiber (1 mm) is much greater than the wavelength of light waves (365 nm);

d) the light delivered into TiO_2-QOFs is composed of rays with the same amount of radiant energy (E_0) and evenly distributed at the fiber cross section;

e) the incident angles (θ) of light range from 0.376π to 0.495π with the increment at 0.0001π.

f) the TiO_2 patchiness on quartz optical fibers, which is defined as the ratio of the surface area of optical fibers in direct contact with TiO_2 nanoparticles to the total surface area of optical fibers, is p (cm^2/cm^2); the average interspace distance between the optical fiber surfaces and the coated TiO_2 layers is z_a (nm).

When light rays with θ between 0.376π to 0.495π approach the interface, each ray activates TiO_2 in the form of evanescent waves if the external substance is air/water or refracted light if the external substance is TiO_2 nanoparticles. For example, when a ray approaches the quartz/water interface, TIR occurs and evanescent waves are thus generated. If TiO_2 is within the penetration depth of evanescent waves (Λ), TiO_2 can be excited by $e^{(-z_a/\Lambda)}$ of radiant energy at this interface while the proportion of $[1-e^{(-z_a/\Lambda)}]$ returns to the optical fiber and continues to propagate as light before the next TIR or refraction spots. Λ is shown in Eq. 12,

$$\Lambda = \frac{\lambda}{4\pi} \cdot \left({n_q}^2 \cdot sin^2\theta - {n_e}^2\right)^{-1/2} \quad (12)$$

where λ is the wavelength of the radiation source, n_q is the refractive index of quartz, and n_e is the refractive index of external medium which must be less than n_q. In contrast, if a ray approaches the interface where TiO_2 is covered (termed quartz/TiO_2 interface), refraction occurs. According to Fresnel equations, most of the light at this interface is transmitted into TiO_2 coating layers from quartz (the fraction of it to the radiant energy at the interface is called transmittance ($T_{q\text{-}TiO2}$), while still a small portion is reflected from the interface to fiber cores for further propagation called reflectance (R) which is equal to (1- $T_{q\text{-}TiO2}$). The $T_{q\text{-}TiO2}$ is be express as Eq. 13,

$$T_{q-TiO2} = 1 - \frac{1}{2}\left\{\left[\frac{n_q \cos\theta - n_T\sqrt{1-\left(\frac{n_q}{n_T}\sin\theta\right)^2}}{n_q \cos\theta + n_T\sqrt{1-\left(\frac{n_q}{n_T}\sin\theta\right)^2}}\right]^2 + \left[\frac{n_q\sqrt{1-\left(\frac{n_q}{n_T}\sin\theta\right)^2} - n_T\cos\theta}{n_q\sqrt{1-\left(\frac{n_q}{n_T}\sin\theta\right)^2} + n_T\cos\theta}\right]^2\right\} \quad (13)$$

where n_T is the refractive index of TiO_2. Summation of the evanescent waves that dissipated into TiO_2 layers ($E_{E,dis}$') and radiant energy refracted into TiO_2 layers ($E_{R,dis}$') at all TIR/refraction spots for all incident ray angles is the radiant energy dissipation (E_{dis}) as shown in Eq. 14.

$$E_{dis} = \sum_{\theta=0.376\pi}^{0.495\pi} E_{E,dis}' + \sum_{\theta=0.376\pi}^{0.495\pi} E_{R,dis}' \quad (14)$$

Supplementary Figure 10 shows the schematic of calculation of $E_{E,dis}$' in TiO_2-QOFs. We assume that the $E_{E,dis}$ at each TIR spot is equal to its radiant energy dissipation of evanescent waves times its probability of TIR. The probability of TIR for single spot is *1-p* according to the surface area of optical fibers not covered by TiO_2. As a result, for a single ray with θ, the $E_{E,dis}$ at the first TIR is equal to $E_0 \cdot e^{-z\alpha/\Lambda} \cdot (1-p)$. For the second TIR spot, there are two cases. One is the first and second spots are both TIR and the other is the first spot is refraction while the second spot is TIR.

The $E_{E,dis}$ at the second TIR spot for the two cases is equal to $E_0 \cdot [(1-e^{za/\Lambda}) \cdot e^{-za/\Lambda}] \cdot (1-p)^2$ and $E_0 \cdot [(1-T) \cdot e^{-za/\Lambda}] \cdot [p \cdot (1-p)]$, respectively. Therefore, the $E_{E,dis}$ at the second TIR is the summation of both, which is $E_0 \cdot e^{-za/\Lambda} \cdot (1-p) \cdot [(1- e^{-za/\Lambda}) \cdot (1-p) + (1-T) \cdot p]$. According to this logic, at the Nth TIR spot (N = 1, 2, 3, …), there are 2^{N-1} cases, and $E_{E,dis}$ at this spot is equal to $E_0 \cdot e^{-za/\Lambda} \cdot (1-p) \cdot [(1- e^{-za/\Lambda}) \cdot (1-p) + (1-T) \cdot p]^N$. The $E_{E,dis}'$ is equal to adding up $E_{E,dis}$ at each TIR spot as shown in Eq. 15.

$$E_{E,dis}' = E_0 \cdot (1-p) \cdot e^{-\frac{z_a}{\Lambda}} + E_0 \cdot (1-p) \cdot e^{-\frac{z_a}{\Lambda}} \cdot \left[\left(1 - e^{-\frac{z_a}{\Lambda}}\right) \cdot (1-p) + (1-\mathrm{T}) \cdot p\right] + \cdots + E_0 \cdot (1-p) \cdot e^{-\frac{z_a}{\Lambda}} \cdot \left[\left(1 - e^{-\frac{z_a}{\Lambda}}\right) \cdot (1-p) + (1-\mathrm{T}) \cdot p\right]^N$$

$$= E_0 \cdot \frac{(1-p) \cdot e^{-\frac{z_a}{\Lambda}} \cdot \left\{1 - \left[(1-p) \cdot (1 - e^{-\frac{z_a}{\Lambda}}) + p \cdot (1 - T_{q-TiO2})\right]^{L/(d \cdot \tan\theta)}\right\}}{(1-p) \cdot e^{-\frac{z_a}{\Lambda}} + p \cdot T_{q-TiO2}} \quad (15)$$

where L is the TiO_2 coating length (cm) and d is the diameter of optical fibers (cm). Based on the same algorithm, we can obtain the total $E_{R,dis}'$, which is shown in Eq. 16.

$$E_{R,dis}' = E_0 \cdot \frac{p \cdot T_{q-TiO2} \cdot \left\{1 - \left[(1-p) \cdot (1 - e^{-\frac{z_a}{\Lambda}}) + p \cdot (1 - T_{q-TiO2})\right]^{L/(d \cdot \tan\theta)}\right\}}{(1-p) \cdot e^{-\frac{z_a}{\Lambda}} + p \cdot T_{q-TiO2}} \quad (16)$$

For our aqueous pollutant degradation study, an LED was attached to a single quartz optical fiber centered in the axial (i.e., longitudinal direction) of a tubular reactor filled with water containing carbamazepine. E_{dis} is a function of E_0, L, d, and n_e, while p and z_a are intrinsic properties of the TiO_2 coating layer and relate only to the coating structure but not the experimental conditions. p and z_a for each TiO_2-QOF can then be calculated by substituting E_0, L, d, n_e, and E_{dis} in air or water obtained by optical measurements when a TiO_2-QOF is surrounded by air or immersed in water. By substituting $E_{dis,air}$ and $E_{dis,water}$ into Eqs. 14–16, p and z_a can be obtained through the 'Solver' function in Microsoft Excel.

D. Derivation of *p* and z_a values.

p and z_a are intrinsic properties of TiO_2 coating layers that related only to the coating structures not the experimental conditions. As a result, their values remain the same regardless of the applied experimental conditions. To obtain values of *p* and z_a, three different conditions (Supplementary Table 1), each changes at a time, were selected. These conditions include: (1) the baseline condition with an irradiance of incident light of 7.02 mW/cm^2 and a TiO_2 coating length of 6.5 cm in air/water as external medium; (2) changing the external medium from air/water to air/ethanol; and (3) changing the TiO_2 coating length from 6.5 to 5.0 cm.

For condition (1), *p* and z_a can be obtained by directly solving both Eqs. 17 and 18 by adding up Eqs. 4 and 5 in air and that in water, respectively.

$$E_{dis,air} = \sum_{\theta=0.376\pi}^{0.495\pi} E_0 \cdot \left\{1 - \left[p \cdot \left(1 - e^{-\frac{z_a}{\frac{\lambda}{4\pi}\cdot\left(n_q{}^2\cdot sin^2\theta - n_{air}{}^2\right)^{-1/2}}}\right) + (1-p)\cdot(1 - T_{q-TiO2})\right]^{\frac{L}{d\cdot tan\,\theta}}\right\} \quad (17)$$

$$E_{dis,water} = \sum_{\theta=0.376\pi}^{0.495\pi} E_0 \cdot \left\{1 - \left[p \cdot \left(1 - e^{-\frac{z_a}{\frac{\lambda}{4\pi}\cdot\left(n_q{}^2\cdot sin^2\theta - n_{water}{}^2\right)^{-1/2}}}\right) + (1-p)\cdot\left(1 - T_{q-TiO2}\right)\right]^{\frac{L}{d\cdot tan\,\theta}}\right\} \quad (18)$$

For condition (2), the change of the external medium of TiO_2-QOFs from water to ethanol indicates that the external refractive index becomes 1.38 instead of 1.33, resulting in the θ_c of TIR changes from 0.376 π to 0.392π. The derivation of $E_{dis,air}$ still follows Eq. 17, while derivation of $E_{dis,ethanol}$ is different from $E_{dis,water}$. The incident light rays with angles from 0.376 π to 0.392π are refracted instead of being totally internal reflected or dissipated as evanescent waves. Besides, the light rays with angles from 0.376π to 0.495π penetrate deeper from the interface as evanescent waves compared with TiO_2-QOFs immersed in water. The transmittance ($T_{q\text{-}ethanol}$) of light ray from quartz to ethanol is expressed as Eq. 19.

$$T_{q-ethanol} = 1 - \frac{1}{2}\left\{\left[\frac{n_q \cos\theta - n_{et}\sqrt{1-\left(\frac{n_q}{n_{et}}\sin\theta\right)^2}}{n_q \cos\theta + n_{et}\sqrt{1-\left(\frac{n_q}{n_{et}}\sin\theta\right)^2}}\right]^2 + \left[\frac{n_q\sqrt{1-\left(\frac{n_q}{n_{et}}\sin\theta\right)^2} - n_{et}\cos\theta}{n_q\sqrt{1-\left(\frac{n_q}{n_{et}}\sin\theta\right)^2} + n_{et}\cos\theta}\right]^2\right\} \quad (19)$$

Where n_{et} is the refractive index of ethanol. The $E_{dis,ethanol}$ is shown in Eq. 20.

$$E_{dis,ethanol} = \sum_{\theta=0.376\pi}^{0.392\pi} E_0 \cdot \left\{1 - \left[p\cdot\left(1 - T_{q-ethanol}\right) + (1-p)\cdot\left(1 - T_{q-TiO2}\right)\right]^{L/(d\cdot\tan\theta)}\right\}$$

$$+ \sum_{\theta=0.392\pi}^{0.495\pi} E_0 \cdot \left\{1 - \left[p\cdot\left(1 - e^{-\frac{z_a}{\frac{\lambda}{4\pi}\cdot\left(n_q{}^2\cdot\sin^2\theta - n_{et}{}^2\right)^{-1/2}}}\right) + (1-p)\cdot\left(1 - T_{q-TiO2}\right)\right]^{L/(d\cdot\tan\theta)}\right\} \quad (20)$$

As a result, p and z_a can be obtained by directly solving Eq. 17, Eq. 19 and Eq. 20.

For condition (3), the decrease in the TiO_2 coating length leads from 6.5 cm to 5 cm allows rays bigger than 0.494π to be directly emitted out from another end of TiO_2-QOFs, resulting in less TIR/refraction occurred. The range of ray angles thus becomes from 0.376π to 0.494π. The p and z should be obtained by solving both Eq. 21 and Eq. 22.

$$E_{dis,air} = \sum_{\theta=0.376\pi}^{0.494\pi} E_0 \cdot \left\{1 - \left[p\cdot\left(1 - e^{-\frac{z_a}{\frac{\lambda}{4\pi}\cdot\left(n_q{}^2\cdot\sin^2\theta - n_{air}{}^2\right)^{-1/2}}}\right) + (1-p)\cdot(1 - T_{q-TiO2})\right]^{\frac{L}{d\cdot\tan\theta}}\right\} \quad (21)$$

$$E_{dis,water} = \sum_{\theta=0.376\pi}^{0.494\pi} E_0 \cdot \left\{1 - \left[p\cdot\left(1 - e^{-\frac{z_a}{\frac{\lambda}{4\pi}\cdot\left(n_q{}^2\cdot\sin^2\theta - n_{water}{}^2\right)^{-1/2}}}\right) + (1-p)\cdot\left(1 - T_{q-TiO2}\right)\right]^{\frac{L}{d\cdot\tan\theta}}\right\} \quad (22)$$

Supplementary Table 2 shows the calculation results of p and z_a values of each TiO_2-QOF under the three different conditions. The average values of p and z_a are 0.528 cm^2/cm^2 and 7.7 nm for TiO_2-QOF-High, 0.206 cm^2/cm^2 and 52.9 nm for TiO_2-QOF-Med, and 0.034 cm^2/cm^2 and 114.3 nm for TiO_2-QOF-Low, respectively, while the variations, as calculated by dividing standard deviation with average values, among p and z_a of each TiO_2-QOF are within 5%.

E. Model verifications

The modeling of light propagation in TiO_2-QOFs makes it possible to predict the radiant energy dissipation in TiO_2-QOFs (E_{dis}) based on their TiO_2 coating layers structures (p and z_a values) and the radiant energy delivered into TiO_2-QOFs (E_0). However, to achieve the purpose of prediction, the accuracy of the model needs to be further verified. In our previous section, p and z_a of their own TiO_2 coating layer structures have been verified to be accurate, but the radiant energy delivered to the TiO_2-QOFs is also critical in predicting the radiant energy dissipation. Therefore, in this Note, the accuracy of the model was further verified by comparing the calculated radiant energy dissipation (Calculated E_{dis}) with the experimental measured radiant energy dissipation (Measured E_{dis}) under different radiant energy delivered into TiO_2-QOFs. To obtain Calculated E_{dis}, the averaged p and z_a values are firstly substituted into the Eq. 17 and Eq. 18, and then adjust the radiant energy delivered into TiO_2-QOFs (E_0) by changing the irradiance of incident light (0.41, 2.04, 4.98 and 7.02 mW/cm^2). The Measured E_{dis} was obtained through directly optical measuring. The verification results are shown in Supplementary Table 3. It is shown that the calculated $E_{dis,air}$ and $E_{dis,water}$ is close to measured ones, and all the error is within 5%, proving the model is accurate.

F. The derivation of the returned radiant energy of evanescent waves and the reflected radiant energy from refraction spots

The returned radiant energy of evanescent waves and the reflected radiant energy from refraction spots are derived based on the ratio of the returned radiant energy of evanescent waves and reflectance on the refraction spots to the total returned radiant energy, respectively. For a single ray with an incident angle between 0.376π and 0.495π, the summation of returned radiant energy

of evanescent waves and the reflected radiant energy from refraction spots (E_{re}) equals to the radiant energy delivered into TiO_2-QOFs (E_0) subtracting the dissipated radiant energy of evanescent waves ($E_{E,dis}'$) and that of refracted light ($E_{R,dis}'$) as shown in Eq. 23.

$$E_{re} = E_0 - E_{E,dis}' - E_{R,dis}' \quad (23)$$

The returned radiant energy is from both the radiant energy of evanescent waves that return to optical fiber if not absorbed by TiO_2 layers, and the reflectance on the refraction spots. The contribution of the returned energy of evanescent waves to the returned radiant energy (C_E) is quantified as the product of the probability of TIR and the ratio of returned evanescent wave energy to the incident light as shown in Eq. 24.

$$C_E = (1-p)\cdot\left(1-e^{-\frac{z_a}{\Lambda}}\right) \quad (24)$$

The contributions of the refracted light (C_R) is quantified as the product of the probability of refraction and the reflectance on the refraction spots as shown in Eq. 25.

$$C_R = p\cdot(1-T) \quad (25)$$

Therefore, their weights to the returned radiant energy is thus equal to their respective contributions divided by the total contribution. The returned radiant energy of evanescent waves ($E_{E,return}$) and the reflected radiant energy from refraction spots ($E_{R,reflect}$) are thus calculated by multiplying the returned radiant energy with their weights of one ray at one angle and then summing up all rays at angles between 0.376π and 0.495π as shown in Eqs. 26 and 27.

$$E_{E,return} = \sum_{\theta=0.376\pi}^{0.495\pi}\left(E_0 - E_{E,dis}' - E_{R,dis}'\right)\cdot\frac{(1-p)\cdot\left(1-e^{-\frac{z_a}{\Lambda}}\right)}{p\cdot(1-T)+(1-p)\cdot\left(1-e^{-\frac{z_a}{\Lambda}}\right)} \quad (26)$$

$$E_{R,reflect} = \sum_{\theta=0.376\pi}^{0.495\pi}\left(E_0 - E_{E,dis}' - E_{R,dis}'\right)\cdot\frac{p\cdot(1-T)}{p\cdot(1-T)+(1-p)\cdot\left(1-e^{-\frac{z_a}{\Lambda}}\right)} \quad (27)$$

G. Relationships between the Λ and z_a

Supplementary Figure 11 shows the relationships between the penetration depth of evanescent waves (Λ) from quartz to water and the average interspace distance between the optical fiber surfaces and coated TiO2 layers (z_a) of the three TiO_2-QOFs. In our system, the incident light angles (θ) is from 0.376π to 0.495π as determined in Supplementary Note 5A. Λ from quartz to water (Λ_{water}) are calculated using Eqs. 1 and 2. When θ is from 0.376π to 0.495π, Λ_{water} shows a big drop from 120 nm to 50 nm. As calculated in Supplementary Note 5D, the average values of z_a are 7.7 nm for TiO_2-QOF-High, 52.9 nm for TiO_2-QOF-Med, and 114.3 nm for TiO_2-QOF-Low. As a result, Λ_{water} is bigger than the value of z_a in TiO_2-QOF-High, suggesting evanescent waves mostly penetrate into the TiO_2 coating layers, and only a small amount of which return to the optical fibers. In the case of TiO_2-QOF-Med, Λ_{water} is bigger than the value of z_a in TiO_2-QOF-Med when θ is from 0.376π to 0.440π but a little lower than z_a in TiO_2-QOF-Med when θ is bigger than 0.440π. The increase in z_a from TiO_2-QOF-High to TiO_2-QOF-Med but the same Λ_{water} suggests more evanescent waves return to TiO_2-QOF-Med than to TiO_2-QOF-High because a portion of evanescent waves cannot reach the TiO_2 coating layer in the TiO_2-QOF-Med. In the case of TiO_2-QOF-Low, Λ_{water} becomes smaller than the value of z_a, giving the most amount of evanescent waves returned to optical fibers.

H. Radiant energy of evanescent waves dissipated into TiO_2 coating layers at different z_a

Supplementary Figure 12 shows percentages of the radiant energy of evanescent waves dissipated into TiO_2 coating layers ($E_{E,dis}'$) to the generated radiant energy of evanescent waves ($E_{E,g}$), at each TIR spot, as a function of light incident angles in the three TiO_2-QOFs at different z_a. As shown in Supplementary Figure 12, all the three TiO_2-QOFs at different z_a show a decreasing trend in the percentage of $E_{E,dis}'$ to $E_{E,g}$ with increasing light incident angles from 0.376π to 0.500π. However, with increasing z_a from 7.7 nm in TiO_2-QOF-High to 114.3 nm in TiO_2-QOF-Low, the percentage of $E_{E,dis}'$ to $E_{E,g}$ at each TIR spot decreases.

I. $E_{E,dis}'$, $E_{R,dis}'$, $E_{E,return}$, and $E_{R,reflect}$ when TiO_2-QOFs were exposed to air

Supplementary Figure 13(a) compares the calculated $E_{E,dis}'$, $E_{R,dis}'$, $E_{E,return}$ and $E_{R,reflect}$ in air against area-specific TiO_2 coating densities. With decreasing area-specific TiO_2 coating densities from TiO_2-QOF-High to TiO_2-QOF-Med and TiO_2-QOF-Low, the generated evanescent wave energy ($E_{E,g}$), i.e., the sum of $E_{E,dis}'$ and $E_{E,return}$, increased from 0.42 to 0.43 and 0.60 J, respectively, and the generated refracted light energy ($E_{R,g}$), i.e., the sum of $E_{R,dis}'$ and $E_{R,reflect}$, decreased from 0.35 to 0.34 and 0.17 J, respectively. The increasing $E_{E,g}$ and decreasing $E_{R,g}$ from TiO_2-QOF-High to TiO_2-QOF-Med and then to TiO_2-QOF-Low confirmed that TiO_2-QOF-Low which has the lowest patchiness TiO_2 promotes Mode 3 of light propagation and generates more evanescent waves.

We further compared the ratios of $E_{R,dis}'$ to $E_{R,g}$ and those of $E_{E,dis}'$ to $E_{E,g}$ in the three TiO_2-QOFs. All ratios of $E_{R,dis}'$ to $E_{R,g}$ were close to 1, confirming that refracted light mostly propagated away from TiO_2-QOFs and its radiant energy dissipated out. However, with increasing $E_{E,g}$ from TiO_2-QOF-High to TiO_2-QOF-Low, the ratios of $E_{E,dis}'$ to $E_{E,g}$ decreased from 0.95 to 0.23. This

is because z_a of TiO_2-QOFs modulates the evanescent wave energy returning to optical fibers. The value of z_a in TiO2-QOF-High (7.7 nm) is much smaller than the value of Λ from quartz to air (30 nm) (Supplementary Figure 13(b)), suggesting a negligible amount of evanescent waves returned to the optical fibers to give a highest ratio of $E_{E,dis}$' to $E_{E,g}$. As increasing z_a from 7.7 nm in TiO_2-QOF-High to 52.9 nm in TiO_2-QOF-Med and 114.3 nm in TiO_2-QOF-Low, more evanescent waves returned to the optical fibers, since they cannot reach the TiO_2 coating layers, thus, to give lower ratios of $E_{E,dis}$' to $E_{E,g}$.

Note 6. Carbamazepine degradation performance

A. Radiant flux dissipation along TiO_2-QOFs

Supplementary Fig. 15a shows the simulated radiant flux dissipated at each coating section at a length of 1 cm along TiO_2-QOFs as calculated from the energy balance model. It shows that 80% of the light launched to TiO_2-QOF-High was dissipated at the first 2-cm section, while only 12% of which was dissipated at the following sections. In contrast, only 43% of the light launched to TiO_2-QOF-Low was dissipated at the first 2-cm section and light was more evenly dissipated along the fiber.

The large amount of light dissipated at the beginning section of TiO_2-QOF-High resulted in local photon oversaturation and caused efficiency losses. This was proved by an additional experiment, which shows the degradation rate constants of carbamazepine and quantum yields by the UV-irradiated TiO_2-QOF-High at a coating length of 1 cm as a function of radiant flux dissipation (Supplementary Fig. 15b). With increasing radiant flux dissipation from 3 to 36 μW, the degradation rate constants of carbamazepine by 1-cm TiO_2-QOF-High increased by 1.8 times, while the quantum yields showed a significant drop by 77%. Therefore, the radiant flux dissipated

at the first 2-cm section of TiO_2-QOF-High at 44 μW contributed to over 75% of carbamazepine degradation but with a low quantum yields of 0.014, while only 25% of carbamazepine degradation was attributed to the last 4-cm of TiO_2-QOF-High. The local photon oversaturation and its high efficiency losses at the first 2-cm section cause the low overall quantum yield of 6-cm TiO_2-QOF-High. In contrast, TiO_2-QOF-Low has a more evenly dissipated radiant flux of 2 to 15 μW, which grant TiO_2-QOF-Low higher quantum yields in degrading carbamazepine.

The novel fiber coating strategy we discovered and reported in this manuscript modulates the coating layer structures to generate higher portions of evanescent waves. The generation of evanescent waves results in even dissipation of light in TiO_2-QOFs and thus prevents local light oversaturation and its associated efficiency losses.

B. Evaluation the difference in mass transfer from different TiO_2 coating layers

We evaluate the impacts of external mass transfer and internal mass transfer of the TiO_2 coating layers on the photocatalytic performance of different TiO_2-QOFs. The external mass transfer describes the diffusion of pollutants from bulk solutions to the TiO_2 coating surface. It is a function of the Reynolds number[1]. Therefore, external mass transfer of the three TiO_2-QOFs are the same because the reactor setup is the same. On the other hand, the internal mass transfer describes the diffusion of pollutants and radicals inside the porous TiO_2 coating layers. It is an intrinsic property of the TiO_2 coatings and determined by the nature of TiO_2 and the coating structures[7,12]. The internal mass transfer could be evaluated using the Thiele modulus (ϕ) and the internal effectiveness factor (η) as shown in Eqs. 28and 29, respectively[12].

$$\phi = \delta\sqrt{\frac{k\tau}{D\varepsilon}} \quad (28)$$

$$\eta = \frac{tanh\,\phi}{\phi} \quad (29)$$

where δ is the TiO_2 coating thickness (m), k is the first-order reaction rate constant (s^{-1}), D is the diffusion coefficient of water (2.3×10^{-9} m^2/s at 20°C[13]), ε is the TiO_2 coating porosity, τ is the TiO_2 coating tortuosity calculated using Eq. 30[14].

$$\tau = \sqrt{\frac{2\varepsilon}{3[1-1.209(1-\varepsilon)^{2/3}]}+\frac{1}{3}} \quad (30)$$

The calculated ϕ and η of the three TiO_2-QOFs are shown in Supplementary Table 4. ϕ of the TiO_2 coating layers decrease from 1.20×10^{-4} in TiO_2-QOF-High to 1.95×10^{-5} in TiO_2-QOF-Low, while all η of the three TiO_2-QOFs equal to 1.00. According to Weisz's criteria, in which the internal mass transfer is neglectable when $\phi < 0.3$ and $\eta \approx 1$[15], the TiO_2 coating layers on the three TiO_2-QOFs have no internal mass transfer limitation.

Therefore, the same external mass transfer and the neglectable internal mass transfer of the TiO_2 coating layers on the three TiO_2-QOFs suggest there is no difference in mass transfer and thus excluding the mass transfer effect on the photocatalytic activity of TiO_2-QOFs.

C. Relationships between *p* and z_a and photocatalytic performance of TiO_2-QOFs

We fabricate two more TiO_2-QOFs, including TiO_2-QOF-Low'' at p of 0.018 and z_a of 139.50 nm and TiO_2-QOF-Low' at p of 0.026 and z_a of 127.97 nm, using different coating conditions as shown in Supplementary Table 5. These two TiO_2-QOFs are then compared with TiO_2-QOF-High, TiO_2-QOF-Med and TiO_2-QOF-Low. As shown in Supplementary Table 5, the carbamazepine degradation rate constants increased with increasing p and decreasing z_a from TiO_2-QOF-Low'' to TiO_2-QOF-Low, but remained the same with further increasing p and decreasing z_a from TiO_2-

QOF-Low to TiO_2-QOF-High. A turning point exists where the carbamazepine degradation rate constants reach a plateau. On the other hand, quantum yields of carbamazepine degradation by the UV irradiated TiO_2-QOF-Low was the highest, at 0.0248. Increasing p and decreasing z_a or decreasing p and increasing z_a resulted in a decrease in quantum yields.

D. Simulation of the rate constant and quantum yield of carbamazepine degradation by TiO_2-QOF-Low at 26 cm coating length

The saved radiant energy in TiO_2-QOF-Low by evanescent waves allows to extend the length of TiO_2-QOF-Low to fully utilize the light energy and increase the photocatalytic reactive sites. As determined earlier, TiO_2-QOF-High has 92.6% of the radiant energy delivering into TiO_2-QOFs dissipated into TiO_2 coating layers. To achieve the ratio of the radiant energy dissipation to the radiant energy delivering into TiO_2-QOFs at 92.6%, the TiO_2 coating length of TiO_2-QOF-Low could be extended to 26 cm which is four times larger than the length in TiO_2-QOF-High (6.5 cm). The length of the new reactor with the 26-cm TiO_2-QOF-Low is thus 4 times larger than that of the original one with the 6.5-cm TiO2-QOF-High. To estimate the performance of the 26-cm TiO_2-QOF-Low, it is considered as four consecutive 6.5 cm segments as shown in Supplementary Figure 16(a). The irradiance emitting from the previous segment is that entering the next one, thus, at an incident light irradiance of 7.02 mW/cm^2 entering the first segment, the irradiance entering each segment is 7.02, 1.92, 1.15 and 0.83 mW/cm^2. To obtain the degradation rate constant (k) under each irradiance in the corresponding segment, the test of carbamazepine at initial concentration of 2 μM degraded by a 6.5-cm TiO_2-QOF-Low in a 23-mL reactor under different irradiances in 4 h was conducted as shown in Supplementary Figure 16(b). The result shows that with the increasing irradiance from 0.41 to 7.02 mW/cm^2, k increased proportionally. As a result, k of the 4 segments

is 0.240, 0.093, 0.070 and 0.061 h^{-1}, respectively, as determined from Supplementary Figure 16(b). The overall degradation rate of the reactor containing 26-cm TiO_2-QOF-Low (dM/dt) is thus determined using Eq. 31,

$$\frac{dM}{dt} = \sum_{i=1}^{4} k_i V[CBZ]_0 \qquad (31)$$

where k_i is the pseudo-first order degradation rate constant of the corresponding segment (h^{-1}), V is the reactor volume of segments (L), $[CBZ]_0$ is the initial concentration of carbamazepine (μM). The quantum yield of the reactor containing 26-cm TiO_2-QOF-Low is determined using Eq. 32,

$$\eta = \frac{\sum_{i=1}^{4} k_i V[CBZ]_0}{I_{dis} \cdot A} \qquad (32)$$

where I_{dis} is the irradiance dissipated in the TiO_2-QOFs (mol-photons/(cm^2·h)), and A is the cross-sectional area of optical fibers (cm^2). If extending the coating length of TiO_2-QOF-High to 26 cm, its calculations of degradation rate and quantum yield also follow Eqs. 31 and 32, but k of the last 3 segments is equal to 0 because light energy is consumed within the beginning 6.5 cm.

The comparison in carbamazepine degradation between the reactor containing the TiO_2-QOF-High at 6.5 cm TiO_2 coating length and the reactor containing the TiO_2-QOF-Low at 26 cm TiO_2 coating length in a 92 mL reactor is shown in Supplementary Figure 16(c). The degradation rate constant of the 26-cm TiO_2-QOF-Low is 0.116 h^{-1}, which is 97% higher than the value of the 6.5-cm TiO_2-QOF-High (0.059 h^{-1}). Besides, the quantum yield of carbamazepine degradation by the 26-cm TiO_2-QOF-Low is 0.039, which is 106% higher than the value by the 6.5-cm TiO_2-QOF-High (0.019).

E. Carbamazepine degradation by UV-irradiated TiO_2-QOF bundles

To demonstrate that the use of the transmitted light by using longer TiO_2-QOF-Low is more efficient than the use of refracted light by using TiO_2-QOF-High bundles, we conducted additional experiments in which seven TiO_2-QOFs were bundled together and used for carbamazepine degradation as shown in Supplementary Figs. 17 and 18. The conclusion was that optimizing evanescent waves to fully use transmitted light in longer TiO_2-QOF-Low is 44–96% more efficient than harvesting refracted light by using TiO_2-QOF-High bundles depending on fiber spacings (1 mm to 7 mm). Meanwhile, TiO_2-QOF-Low uses 77% fewer photocatalysts than TiO_2-QOF-High. Below are details on these additional, new experiments which are integrated into the manuscript.

As shown in Supplementary Fig. 17a, a hexagonal arrangement for a TiO_2-QOF bundle which consists of one TiO_2-QOF in the center and six TiO_2-QOFs at the edge was proposed. The hexagonal arrangement is the closest packing that allows TiO_2-QOFs to utilize the most refracted light out of fibers. The minimum distance between two TiO_2-QOF surfaces, either from the TiO_2-QOF in the center to the ones at the edge or between those at the edge, are defined as the fiber spacing (S) in the bundle. The S was set as 1, 3, 5 and 7 mm in the supplementary tests. The TiO_2-QOF bundle was installed in a tubular reactor of an inner length of 65 mm and an inner diameter of 24 mm. All seven TiO_2-QOFs have a coating length of 6.5 cm. Light was only allowed to be launched from a UV-LED to the TiO_2-QOF in the center of the bundle, i.e., the one centered in the axial of the reactor.

Supplementary Fig. 17b shows the comparisons in carbamazepine degradation rates (r) by a single fiber of TiO_2-QOF-High, a single fiber of TiO_2-QOF-Low, a bundle of TiO_2-QOF-High, and a bundle of TiO_2-QOF-Low irradiated by one UV-LED at a UV intensity of 7.02 mW/cm^2. The S in the two bundles were 1 mm. The carbamazepine degradation rate by a 6.5-cm TiO_2-QOF-

High ($r_{c\text{-}High}$) was 0.0065 μmole h^{-1}. Under the same experimental condition, the carbamazepine degradation rate by a 6.5 cm TiO_2-QOF-Low was also 0.0065 μmole h^{-1}. But to fully utilize the incident light launched into the single TiO_2-QOF-Low, it shall be extended to 26 cm. The 26 cm TiO_2-QOF-Low thus showed a carbamazepine degradation rate ($r_{c\text{-}Low}$) of 0.0127 μmole h^{-1}, which was 97% higher than $r_{c\text{-}High}$ (Manuscript Fig. 5d). By bundling seven 6.5-cm TiO_2-QOF-High together using our proposed hexagonal arrangement, the carbamazepine degradation rate by a bundle of TiO_2-QOF-High (r_{High}) was 0.0109 μmole h^{-1}, which was 67% higher than $r_{c\text{-}High}$. Moreover, by bundling seven 26-cm TiO_2-QOF-Low, the carbamazepine degradation rate was further improved to 0.0148 μmole h^{-1}, as calculated by adding up the carbamazepine degradation rates by a 6.5-cm TiO_2-QOF-Low bundle and that by the extended 19.5 cm portion of the 26-cm TiO_2-QOF-Low in the center. The degradation rate by a 26-cm TiO_2-QOF-Low bundle (r_{Low}) of 0.0148 μmole h^{-1} is thus about 1.36 times as high as r_{High}. In TiO_2-QOF bundles, r was attributed to one TiO_2-QOF in the center irradiated directly by a UV-LED and six TiO_2-QOFs at the edge irradiated by refracted light from the one in the center. Therefore, r_{High} and r_{Low} can be expressed as Eqs. 1 and 2, respectively.

$$r_{High} = r_{c-High} + 6r_{e-High} \quad (1)$$

$$r_{Low} = r_{c-Low} + 6r_{e-Low} \quad (2)$$

where $r_{e\text{-}High}$ and $r_{e\text{-}Low}$ are the carbamazepine degradation rate attributed to each of the six TiO_2-QOF-High at the edge and each of the six TiO_2-QOF-Low at the edge, respectively. Besides, since all the four TiO_2-QOF systems received the same UV intensity, their apparent quantum yields followed the same trend as their degradation rates.

As the S affects the amount of refracted light received by the TiO_2-QOFs at the edge and thus the carbamazepine degradation rate by a TiO_2-QOF bundle. r as a function of S were examined

and shown in Supplementary Fig. 17c using the above-mentioned experimental setup. With an increase in S from 1 to 7 mm, both r_{High} and r_{Low} decreased proportionally. By subtracting $r_{c\text{-}High}$ from r_{High} and $r_{c\text{-}Low}$ from r_{Low}, $r_{e\text{-}High}$ and $r_{e\text{-}Low}$ as a function of S were obtained as shown in Eqs. 3 and 4, respectively. Eqs. 3 and 4 suggest both $r_{e\text{-}High}$ and $r_{e\text{-}Low}$ decrease with increasing S.

$$r_{e-High} = \frac{-0.0007S + 0.005}{6} \quad (3)$$

$$r_{e-Low} = \frac{-0.0003S + 0.0023}{6} \quad (4)$$

However, in practice, light will be delivered to all TiO_2-QOFs in the bundle but not just to a single TiO_2-QOF. Therefore, the carbamazepine degradation rates by (i) a bundle of TiO_2-QOF-Low consisting of seven 26-cm TiO_2-QOF-Low each irradiated by one UV-LED (r'_{Low}), and (ii) a bundle of TiO_2-QOF-High consisting of seven 6.5-cm TiO_2-QOF-High each irradiated by one UV-LED (r'_{High}), were simulated using the data we obtained from Supplementary Fig 17c. The TiO_2-QOF in the center receives light launched from a UV-LED and refracted light from the surrounding six TiO_2-QOFs at $S = x$ (Supplementary Fig. 18a). Each of the six TiO_2-QOFs at the edge receives light launched from a UV-LED, refracted light from the surrounding three TiO_2-QOFs at $S = x$, and refracted light from two TiO_2-QOFs at a further distance $S' = [(x+1)3^{0.5}-1]$ (Supplementary Fig. 18b). Therefore, the overall carbamazepine degradation rate of seven TiO_2-QOFs each irradiated by one UV-LED (r') were calculated by summing the carbamazepine degradation rate by each of the seven TiO_2-QOFs as shown in Eq. 5.

$$r' = \left(r_c + 6r_{e\,|\,S=x}\right) + 6\left(r_c + 3r_{e\,|\,S=x} + 2r_{e\,|\,S'=\sqrt{3}(x+1)-1}\right) \quad (5)$$

The r'_{Low} and r'_{High} as a function of S were then calculated and shown in Supplementary Fig. 18c. Both r'_{Low} and r'_{High} were the highest at an S of 1 mm. At such a small S, r'_{Low} is 44% higher than r'_{High}. This result shows TiO_2-QOF-Low which generates high quantity of evanescent waves

to activate TiO_2 is more efficient to degrade carbamazepine compared with TiO_2-QOF-High even when they are bundled together. Nonetheless, such compact arrangement of TiO_2-QOFs with an *S* of 1 mm cannot guarantee uniform mixing as the reactor scales up when installing more TiO_2-QOFs and may compromise the degradation rates and apparent quantum yields. A larger *S* is thus required. With increasing *S* from 1 to 7 mm, the differences between r'_{Low} and r'_{High} increase from 44% to 96% (Supplementary Fig. 18c). The advantage of TiO_2-QOF-Low becomes more significant compared with TiO_2-QOF-High at larger *S*. Besides, a 26-cm TiO_2-QOF-Low bundle uses 77% fewer photocatalysts compared with that used in a 6.5-cm TiO_2-QOF-High bundle.

These results show that optimizing evanescent waves to fully use transmitted light in longer TiO_2-QOF-Low is 44–96% more efficient than harvesting refracted light by using TiO_2-QOF-High bundles depending on fiber spacings (1 mm to 7 mm). By controlling surface patchiness and distance between fiber surface and photocatalyst coating layers, TiO_2-QOF-Low not only prevents light oversaturation and its associated efficient losses (as demonstrated in Comment 2), but also reduces light wasted by refraction and increase surface reactive sites. These features make TiO_2-QOF-Low more energy-efficient to degrade pollutants.

F. TiO_2 coating stability tests

TiO_2 coating is stable during the reaction. Tests are conducted to evaluate the radiant energy dissipation and carbamazepine degradation rate constants of TiO_2-QOF-Low for 3 cycles. For each cycle, TiO_2-QOF-Low was immersed in the carbamazepine containing solution under stirring for 4 h. After each cycle, the used TiO_2-QOF-Low was dried before running the next cycle. Supplementary Fig. 19 shows that both radiant energy dissipation and carbamazepine degradation

rate constants remain unchanged for the 3 cycles of testing, suggesting TiO_2 coating is stable during the reaction.

Supplementary Methods

A. Preparation of uncoated quartz optical fibers.

Quartz optical fibers (FT1000UMT; numerical aperture of 0.39, Ø1000 μm Core Multimode Optical Fiber, High-OH for 300−1200 nm) and polishing/stripping equipment were purchased from Thorlabs. The uncoated optical fibers were prepared by removing the buffer coating using a stripping tool (M44S63), and then were cut into segments of specified lengths using a tungsten steel blade. The segments were soaked in acetone for 24 hours, then 1 M KOH for another 24 hours to remove the TECS cladding, and finally washed with DDI water. Both tips of the uncoated optical fibers were polished using a polishing assembly (D50SMA) with LF30P, LF5P and LF03P fiber polishing paper in that order. The clarity of both tips was critically examined using a fiber inspection scope (FS201) to ensure a good light transmission.

B. Preparation of cross-section samples of TiO_2-QOFs using Focused Ion Beam (FIB)

The purpose of using Focused Ion Beam (FIB) in this study is to prepare cross-section samples of TiO_2-QOFs for transmission electron microscopy (TEM) using an in-situ lift-out technique[11]. To achieve this, a Helios G4 UX Dual-Beam FIB/SEM System containing both a focused Ga+ ion beam and an ultra-high resolution field emission scanning electron column was applied. In this method, the prepared segments of TiO_2-QOFs were firstly placed on a sample stub and then coated with an Au layer on their surfaces by a gold coater (Scancoat Six, Edwards) in order to capture images during the further operation, and then the sample stub was transferred to the chamber of the FIB machine. The preparation procedures are shown in Supplementary Fig. 20. Initially, a thin Pt protection layer (8 μm × 1.5μm × 400 nm) was deposited on the surfaces of TiO_2-QOFs by an electron-beam in case that the TiO_2 layers are sensitive to ion-beam. Then, a thicker Pt protection

layer was covered on the thin Pt layer by an ion beam in a size about 10 μm × 2 μm × 1.5 μm. In the next step, two opposing trenches were roughly milled away, leaving a thin cross section of thickness at around 1.5 μm on the block sample to form a pre-lamella, after which cleaning both sides of the pre-lamella. The next step is called U-cut, which involves cutting the bottom and the side of the pre-lamella, and leaving only a branch point on the bulk sample to fix the pre-lamella. To lift off this cut pre-lamella, an EasyLift needle was inserted and then welding the pre-lamella to the EasyLift needle by ion beam-assisted Pt deposition. After cutting away the branch point, the pre-lamella could be lifted off by retracting the needle, before being transferred and then welded to a TEM grid. Subsequently, using low incident angles and low currents of ion beams to finish final thinning and polishing procedures until the pre-lamella down to a thickness of less than 100 nm, so that the TEM lamella of cross-section of TiO_2-QOFs was completed.

Supplementary References

1. Hui, R. & O'Sullivan, M. Fundamentals of Optical Devices. *in Fiber Optic Measurement Techniques.* 1–128 (Academic Press, 2009).

2. Peatross, J. & Michael, W. *Physics of Light and Optics. Physics of Light and Optics.* (Brigham Young Univ. Press, Provo, 2017).

3. Axelrod, D. Total Internal Reflection Fluorescence Microscopy in Cell Biology. *Traffic* **2,** 764–774 (2001).

4. Sarkar, A., Robertson, R. B. & Fernandez, J. M. Simultaneous atomic force microscope and fluorescence measurements of protein unfolding using a calibrated evanescent wave. *Proc. Natl. Acad. Sci. U.S.A.* **101,** 12882–12886 (2004).

5. Lensun, L., Smith, T. A. & Gee, M. L. Partial Denaturation of Silica-Adsorbed Bovine Serum Albumin Determined by Time-Resolved Evanescent Wave-Induced Fluorescence Spectroscopy. *Langmuir* **18,** 9924–9931 (2002).

6. Chen, Y. et al. Study on the propagation mechanism of evanescent waves in one-dimensional periodic photonic crystal. *Phys. Lett. A* **379,** 2257–2260 (2015).

7. Chen, D., Li, F. & Ray, A. K. Effect of mass transfer and catalyst layer thickness on photocatalytic reaction. *AIChE J.* **46,** 1034–1045 (2000).

8. Sun, J. et al. The influence of the UV/chlorine advanced oxidation of natural organic matter for micropollutant degradation on the formation of DBPs and toxicity during post-chlorination. *Chem. Eng. J.* **373,** 870–879 (2019).

9. Pan, Y. et al. UV/chlorine treatment of carbamazepine: Transformation products and their formation kinetics. *Water Res.* **116,** 254–265 (2017).

10. Keen, O. S., Baik, S., Linden, K. G., Aga, D. S. & Love, N. G. Enhanced biodegradation of carbamazepine after UV/H_2O_2 advanced oxidation. *Environ. Sci. Technol.* **46,** 6222–6227 (2012).

11. Sezen, M. Focused Ion Beams (FIB) — Novel Methodologies and Recent Applications for Multidisciplinary Sciences. *in Modern Electron Microscopy in Physical and Life Sciences*. (IntechOpen Press, London, 2016).

12. Visan, A., Van Ommen, J. R., Kreutzer, M. T. & Lammertink, R. G. H. Photocatalytic reactor design: Guidelines for kinetic investigation. *Ind. Eng. Chem. Res.* **58,** 5349–5357 (2019).

13. Barnes, C. J. & Turner, J. V. Isotopic Exchange in Soil Water. *in Isotope Tracers in Catchment Hydrology*. (Elsevier, 1998).

14. Ahmadi, M. M., Mohammadi, S. & Hayati, A. N. Analytical derivation of tortuosity and permeability of monosized spheres: A volume averaging approach. *Phys. Rev. E* **83,** 026312 (2011).

15. Doran, P. M. Heterogeneous Reactions. in Bioprocess Engineering Principles. (Elsevier, 2013).
